# Metagenomic insights into the development of microbial communities of straw and leaf composts

**DOI:** 10.3389/fmicb.2024.1485353

**Published:** 2025-01-22

**Authors:** Anastasiia K. Kimeklis, Grigory V. Gladkov, Olga V. Orlova, Tatiana O. Lisina, Alexey M. Afonin, Tatiana S. Aksenova, Arina A. Kichko, Alla L. Lapidus, Evgeny V. Abakumov, Evgeny E. Andronov

**Affiliations:** ^1^Laboratory of Microbiological Monitoring and Bioremediation of Soils, All-Russian Research Institute of Agricultural Microbiology, Saint Petersburg, Russia; ^2^Department of Applied Ecology, Saint-Petersburg State University, Saint Petersburg, Russia; ^3^A.I. Virtanen Institute for Molecular Sciences, University of Eastern Finland, Kuopio, Finland; ^4^Independent Researcher, Saint-Petersburg, Russia

**Keywords:** straw, leaf litter, soil of Chernevaya taiga, cellulose decomposition, PUL, MAG, amplicon sequencing, metagenome

## Abstract

**Introduction:**

Soil microbiome is a major source of physiologically active microorganisms, which can be potentially mobilized by adding various nutrients. To study this process, a long-term experiment was conducted on the decomposition of oat straw and leaf litter using soil as a microbial inoculum.

**Methods:**

Combined analyses of enzymatic activity and NGS data for 16S rRNA gene amplicon and full metagenome sequencing were applied to study taxonomic, carbohydrate-active enzyme (CAZy), and polysaccharide utilization loci (PULs) composition of microbial communities at different stages of decomposition between substrates.

**Results:**

In straw degradation, the microbial community demonstrated higher amylase, protease, catalase, and cellulase activities, while peroxidase, invertase, and polyphenol oxidase were more active in leaf litter. Consistent with this, the metagenome analysis showed that the microbiome of straw compost was enriched in genes for metabolic pathways of simpler compounds. At the same time, there were more genes for aromatic compound degradation pathways in leaf litter compost. We identified nine metagenome-assembled genomes (MAGs) as the most promising prokaryotic decomposers due to their abnormally high quantity of PULs for their genome sizes, which were confirmed by 16S rRNA gene amplicon sequencing to constitute the bulk of the community at all stages of substrate degradation. MAGs from Bacteroidota (*Chitinophaga* and *Ohtaekwangia*) and Actinomycetota (*Streptomyces*) were found in both composts, while those from Bacillota (*Pristimantibacillus*) were specific for leaf litter. The most frequently identified PULs were specialized on xylans and pectins, but not cellulose, suggesting that PUL databases may be underrepresented in clusters for complex substrates.

**Discussion:**

Our study explores microbial communities from natural ecosystems, such as soil and lignocellulosic waste, which are capable of decomposing lignocellulosic substrates. Using a comprehensive approach with chemical analyses of the substrates, amplicon, and full metagenome sequencing data, we have shown that such communities may be a source of identifying the highly effective decomposing species with novel PULs.

## 1 Introduction

Majority of the organic carbon storage in terrestrial ecosystems is located in the soil and plants, where it is involved in the global carbon cycle between living and dead biomass, atmosphere, and geosphere (Kayler et al., 2017). Annually, several hundred billion tons of lignocellulosic biomass in the form of agricultural and forestry residues, food, and industrial wastes are produced (Singh et al., 2022; Ojo, 2023). Thus, its decomposition is a major part of organic carbon circulation in terrestrial biomes (Liao et al., 2022). Recently, plant residue decomposition became the focus of metagenomic studies, but due to the high diversity of types of lignocellulosic substrates, the process is highly variable. Thus, the specific features of a particular plant waste decomposition are still insufficiently highlighted (Huang et al., 2023). While the recirculation of plant litter in agricultural ecosystems receives increasing attention (Sun et al., 2023; Liu et al., 2023; Wang et al., 2022), the knowledge about biomass turnover in natural ecosystems remains limited (Giweta, 2020). Yet, leaf litter, as a major component of the annual forest litterfall (Thakur et al., 2022) and the primary component of soil formation, takes longer periods to decompose than straw, a part of agricultural waste, which is linked to the differences in their chemical composition (Manzoni, 2017; Liu et al., 2022).

Lignocellulosic biomass is a complex substrate, mainly consisting of recalcitrant cellulose, hemicellulose, and lignin in varying proportions (Toushik et al., 2017; Chen, 2014). Majority of these compounds are degraded by enzymes, encoded by genes united in the CAZy database, including families of glycoside hydrolases (GHs), glycosyltransferases (GTs), polysaccharide lyases (PLs), carbohydrate esterases (CEs), auxiliary activities (AAs), and carbohydrate-binding modules (CBMs) (Drula et al., 2022). These genes are often combined in gene clusters, providing a full decomposition of a carbohydrate polymer (Zhang et al., 2018). Historically, Bacteroidota exhibited a cluster organization (Bjursell et al., 2006), which was referred to as polysaccharide utilization loci (PUL), which represented the set of physically linked genes organized around a *sus*CD gene pair (Terrapon et al., 2015). Later, cluster organization was shown for other phyla, not obligatorily linked with these genes (Terrapon et al., 2018). Therefore, for the automatic search of such structures, CAZyme gene clusters (CGC) were proposed, which encode at least one CAZyme, one transporter, one transcriptional regulator, and one signaling transduction protein (Zhang et al., 2018). Still, while PULs are curated and experimentally accredited working gene clusters with known substrates, CGCs are not. Thus, PULs are highly sought after in natural ecosystems, but they are mostly described for gut microbiota (Hao et al., 2021). The most frequent carriers of PULs belong to Bacteroidota and Bacillota (Grondin et al., 2017). Whether PULs are limited to these environments and phyla is unclear.

In natural ecosystems, the initial sequestration of carbon is accomplished by soil microorganisms (Wu et al., 2024). Soils represent a global diversity storage of microbiota capable of decomposing lignocellulosic substrates. It was shown that communities from carbon-rich soils are more capable of assimilating organic matter (Nayak et al., 2022). Thus, such soils can be used as a source for the mobilization of active microbiota. For this, we have chosen the soil of Chernevaya taiga (Tomsk, Russia), which forms in the mid-mountainous area in the conditions of low eluviation of nutrients and low erosion (E. V. Abakumov et al., 2020). The unique combination of environmental factors leads to the gigantism of the perennial grass cover (Kravchenko et al., 2022; Achat et al., 2013), which annually leaves up to 400 g/m^2^ of lignocellulosic biomass to be processed by soil microorganisms. This results in the accumulation of the thick humic layer, reaching up to 70 cm in depth. The microbiota from this soil has great potential in decomposing cellulosic substrates, but this issue has not been studied before. Hence, the goal of our study was to reveal this potential by setting up a long-term decomposing experiment, where the soil of Chernevaya taiga was enriched with two contrasting lignocellulosic substrates (straw and leaf litter) to reveal which parts of the microbial community would be more advanced in the new conditions and to reveal differences in the functional potential of these communities. Two substrates of totally different hemicellulose composition were chosen to see to what extent different microbial communities would be mobilized from one soil microbiome under the influence of different substrates. This process was monitored by evaluating the shift of chemical composition of the substrates, taxonomic composition, and metagenome composition of the decomposing microbial communities with a focus on CAZy and PULs organization. The combination of Illumina sequencing of the 16S rRNA gene and Oxford Nanopore metagenome sequencing was used to reveal the most probable active bacterial members of these communities.

## 2 Materials and methods

### 2.1 Experiment setup

**Figure 1:**
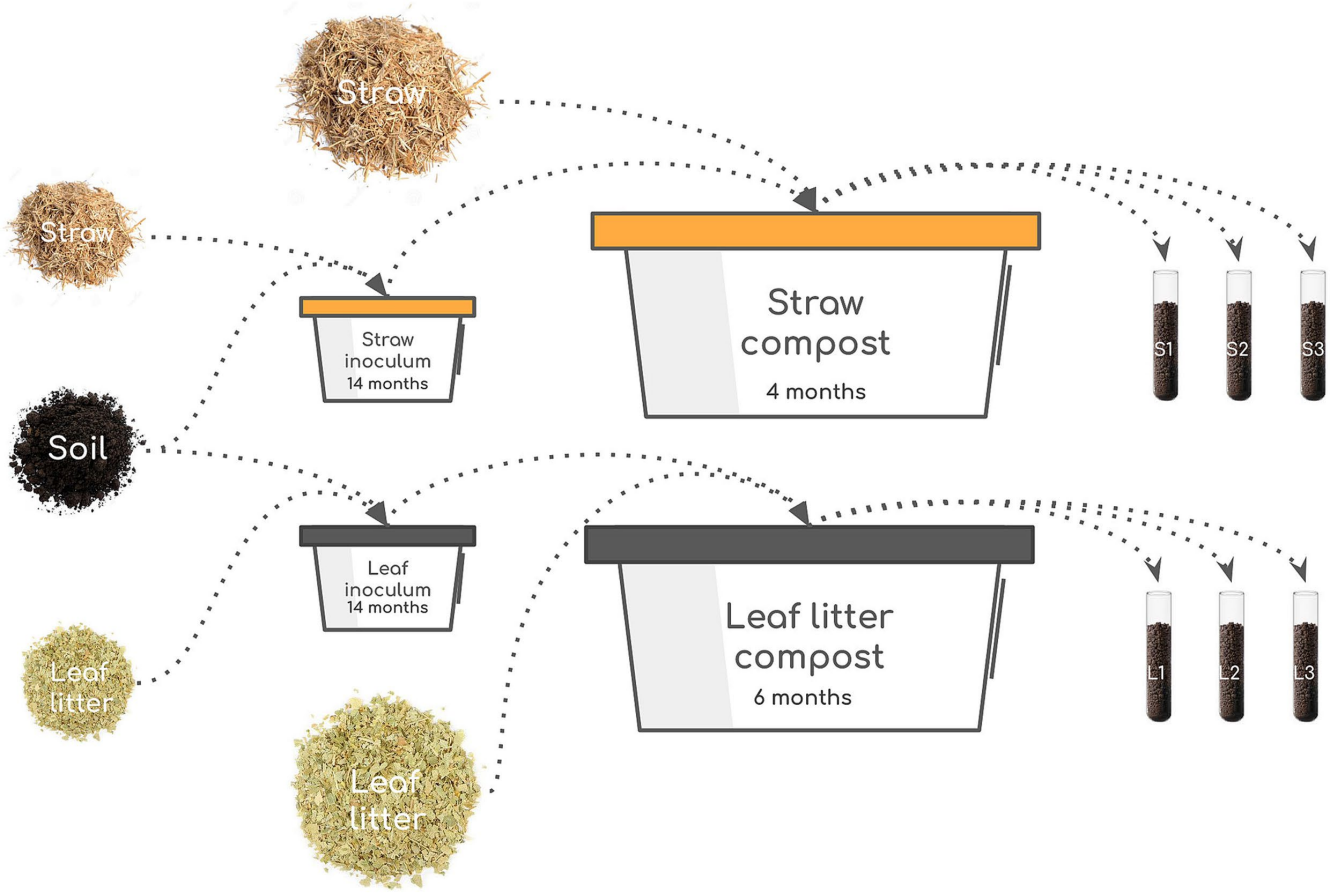
Scheme of the experiment. The starting culture was prepared by mixing soil and substrate (straw or leaves) and incubating for 14 months. The resulting inoculum was mixed with the fresh batch of substrates and incubated for 4 months (straw) or 6 months (leaves). Three samples were taken from each compost: 9, 61, and 111 days for straw and 9, 111, and 174 days for leaves.

As a model of the decomposition of two contrasting cellulosic substrates, we set up the experiment of composting oat straw and leaf litter with the soil-based inoculum (Figure 1).

The soil of Chernevaya taiga was collected near Tomsk, Russia (N 56.30693, E 85.47063) in 2019. The forest is formed by a stable population of *Populus tremula* and *Abies sibirica*. The forbs are comprised of Siberian and Asia-specific tall-herbaceous perennials—*Euphorbia lutescens*, *Saussurea latifolia*, *Heracleum dissectum*, and *Alfredia cernua*. The soil type is Umbric Retisol, and its chemical characteristics and microbial composition were reported earlier (E. V. Abakumov et al., 2020; Kravchenko et al., 2022; Polyakov et al., 2022; Abakumov et al., 2023). Briefly, the topsoil was slightly acidic (pH = 5.8–6.2), with high organic carbon (2.4–9.9%) and nitrogen (0.2–0.6%) content. The phosphorus content reached 1,000 mg/kg, comparable to the soils from tropical rainforests—one of the most productive ecosystems (Reed et al., 2011). Two starter inoculates for the main experiment were prepared by making enrichment cultures by adding cellulose substrates to the soil twice to activate and later increase the number of cellulolytic microorganisms. For this, 500 g of topsoil was mixed with 25 g (20:1 in mass, 1:1 in volume) of air-dry substrates (shredded oat straw or birch leaf litter) in 1-l containers and incubated in a thermostat at 28 ± 2°C with a moisture content of 60%. After 8 months, an additional 12.5 g of the substrates were mixed in. The resulting composts were used as starter inoculates for the main experiment to accelerate the decomposition process.

For the experiment, two types of lignocellulosic biomass were used: (1) oat straw and (2) a mixture of fallen leaves of birch, oak, aspen, and willow (“leaf litter”). The chemical characteristics of the substrates are given in Supplementary Table S1. The substrates were shredded into particles 1–3 cm in size. For each type of lignocellulosic biomass, 3 kg of air-dry substrate, 300 g of compost inoculum, and 9 L of water were mixed in a 60-l polypropylene tub. Nine nylon bags with 5 g of air-dry substrate were placed inside the mixed mass for subsequent assessment of mass loss during composting. The top surface of the composted substrates was covered with plastic film to prevent drying out. Composting was carried out at a constant temperature of 28 ± 2°C in a thermostat room. Moisture content was controlled once a week, and the substance was stirred once every 2 weeks. Important to note is that while the decomposition of lignocellulosic biomass in the soil is accomplished both by fungi and bacteria, the conditions of our experiment favored the growth of the prokaryotic component more than the eukaryotic component since we aimed at exploring the genomic structure of the bacterial decomposers.

Sampling was carried out three times during the experiment based on the external signs of the decomposition stage, which differed for the two substrates: straw on days 9, 61, and 111 and leaf litter on days 9, 111, and 174, for a total of six samples. Furthermore, these dates are referred to as the early phase (day 9 for straw and leaf litter), middle phase (day 61 for straw and day 111 for leaf litter), and late phase (day 111 for straw and day 174 for leaf litter). The chemical analyses of samples were performed immediately after collection, while samples for enzymatic and molecular analyses were stored at −20°C until the end of the experiment.

### 2.2 Sample processing

As a measure of the decomposition process, we evaluated mass loss of the substrate, respiration rates, chemical composition, and enzyme activity for six collected samples. The amount of the decomposed substrate was quantified by measuring the dry weight of the contents of nylon bags at the time of sample collection. The activity of the microbial biomass was assessed through the respiration of the substrate, expressed in the amount of the release of carbon dioxide using the alkali absorption method (Cleve et al., 1979). Enzymatic analyses included amylase, catalase, exocellulase, endocellulase, peroxidase, polyphenol oxidase, invertase, and protease activity (Khaziev, 1976), which were carried out at the Agrophysical Research Institute. Other chemical analyses included the determination of pH, total carbon, nitrogen, cellulose, hemicellulose, and humic compound content (Tsyplenkov et al., 1997; Sharkov et al., 1976), which were conducted both for the samples of composts and of substrates before the decomposition. The pH values were measured using pH meter F690 (Beckman Coulter, Inc., United States). The content of organic carbon (TOC) was expressed through the ash content in the substrates. The content of total nitrogen (TN) was measured on an automatic nitrogen analyzer (Boshu, Switzerland).

For the amplicon sequencing, total DNA was extracted from the six experimental samples in triplicate using the RIAM protocol (Pinaev et al., 2022). Pair-ended libraries were prepared using primers F515 (5’-GTGCCAGCMGCCGCGGTAA-3′) and R806 (5’-GGAC TACVSGGGTATCTAAT-3′) (Bates et al., 2011) for the 16S rRNA gene and ITS3 (5’-GCATCGATGAAGAACGCAGC-3′) and ITS4 (5’-TCCTCCGCTTATTGATATGC-3′) (White et al., 1990) for the ITS2 fragment and sequenced on the Illumina Miseq platform (Illumina, Inc., United States).

DNA for the metagenome sequencing was extracted from the same six experiment samples, pre-stirred in liquid nitrogen, using the NucleoSpin® Soil Kit (Macherey-Nagel GmbH & Co. KG, Germany). The metagenome libraries were prepared using the Ligation Sequencing Kit 1D (Oxford Nanopore Technologies, United Kingdom) and sequenced on the MinION platform with the Flow Cell 9.4.1 rev D (Oxford Nanopore Technologies, Oxford, United Kingdom).

### 2.3 Data analysis

The amplicon sequencing data were processed in Rstudio (R Core Team, 2024) using the DADA2 pipeline (Callahan et al., 2016) as described earlier (Kimeklis et al., 2023), including analysis of alpha-diversity (observed and Shannon (Shannon and Weaver, 1949) indices) and beta-diversity (NMDS (Kruskal, 1964) with Bray–Curtis distances (Bray and Curtis, 1957)). Taxonomic identification of isolated ASVs (amplicon sequencing variants) was performed using Silva 138.1 (for 16S rRNA gene) (Quast et al., 2013) and Unite ver8_02.02.2019 (for ITS2 fragment) (Nilsson et al., 2019) databases. The canonical correspondence analysis (CCA) (Braak et al., 1995) was performed to connect the microbiome composition of the composting substrates with the variability of their chemical characteristics. The data analyses of amplicon sequencing and analysis of chemical characteristics were performed using vegan (Oksanen et al., 2012), phyloseq (McMurdie and Holmes, 2013), and ANCOM-BC (H. Lin and Peddada, 2020) packages.

Metagenomes of the microbial decomposer communities of straw and leaf litter were basecalled by Guppy (Wick et al., 2019), assembled with Flye 2.9 using modifiers meta-and nano-raw (Kolmogorov et al., 2020), and polished with medaka (Medaka, 2024). The program kraken2 (Wood et al., 2019) in combination with the taxonkit utility (Shen and Ren, 2021) was used to determine the taxonomic composition of the metagenomes. The GTDB 214 database (Parks et al., 2022) was used as a reference base for prokaryotes (using the kraken2 struo2 database creation utility), and the PlusPF database based on the RefSeq NCBI database was used to identify eukaryotes. The results obtained were combined with the GTDB database, being prioritized for prokaryote taxonomy divergence. The functional annotation of the metagenomes was performed using EggNOG-mapper v2 (Cantalapiedra et al., 2021) via Diamond with the --frameshift parameter (Buchfink et al., 2021). The annotation results were normalized by the sequencing depth, acquired by minimap2 (Li, 2018) and samtools (Li et al., 2009), and contigs with length less than 10,000 were filtered out. The differences in gene composition between metagenomes of different substrates were assessed using differential expression analysis in limma/voom from the edgeR library (Law et al., 2014). Significantly different KO (KEGG Orthology) identifiers (Kanehisa et al., 2016) between substrates were further processed using the MinPath (Ye and Doak, 2009) program. DRAM annotator data were used to select genes responsible for lignocellulosic substrate decomposition based on functional annotation.

MAGs were isolated from the metagenome assemblies using Semibin2 software (Pan et al., 2023). After polishing the obtained draft bins by the medaka program, the bins were filtered by quality [medium (Genome QC Criteria, 2024) and high (Bowers et al., 2017)] using the CheckM2 program. Taxonomic annotation of MAGs was performed using GTDB-Tk (Chaumeil et al., 2022). PUL search in MAGs was performed using dbCAN in dbCAN-PUL (Ausland et al., 2021). The rRNA genes were isolated from the MAGs using the Barrnap program (Seemann, 2013) and aligned to the amplicon sequencing results. The results were visualized in the R software environment.

The package versions and scripts used in the study are available at the repository https://github.com/crabron/clusters.

## 3 Results

### 3.1 The chemical characteristics of the composting process

**Figure 2:**
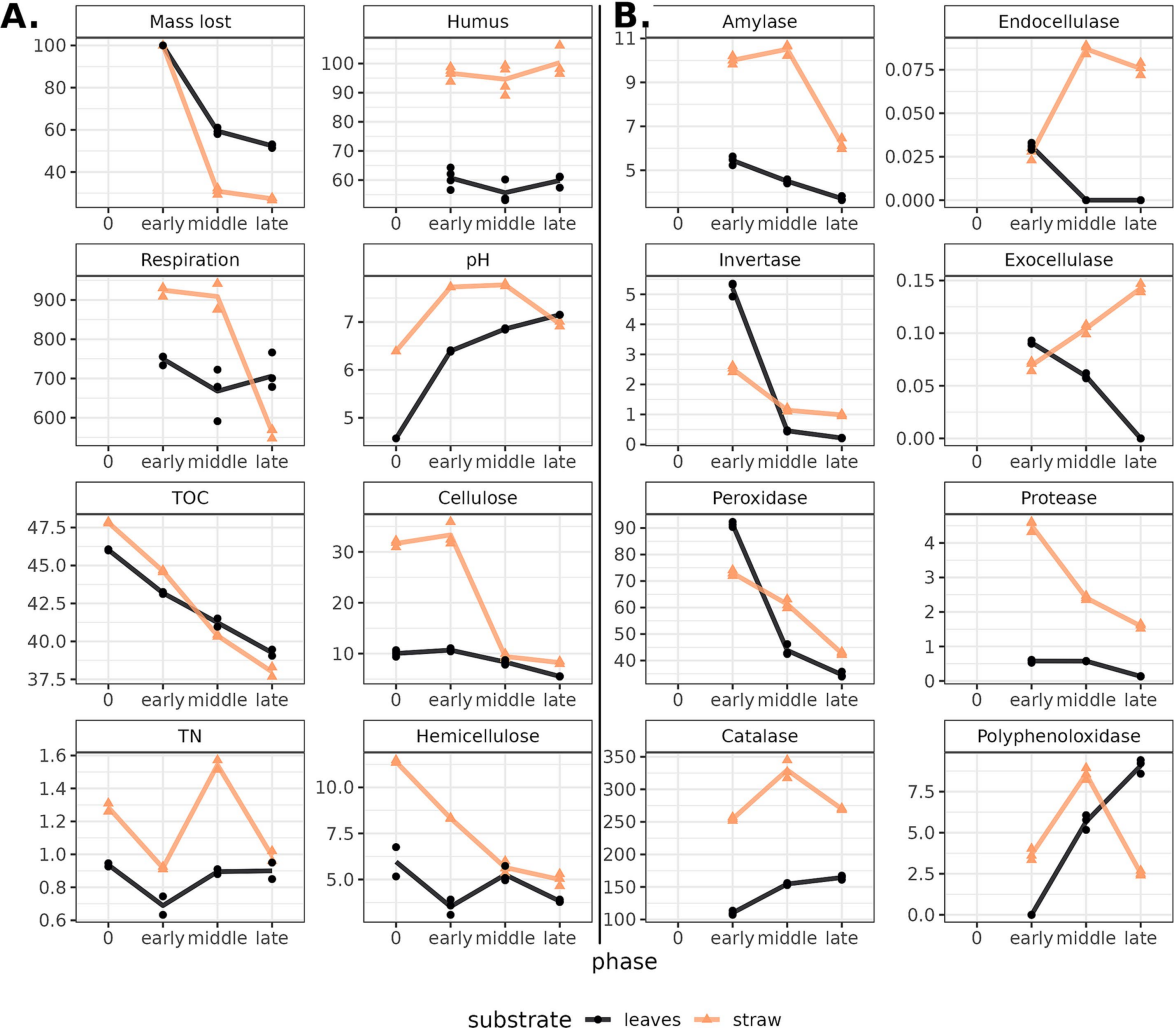
**(A)** Chemical characteristics and **(B)** enzyme activity in composts (leaves—black line, straw—yellow) during decomposition. Phase “0” stands for the characteristics of the cellulosic substrate without the soil-based inoculum. The Y-axis corresponds with the absolute values of the parameters (see Supplementary Table S1). Dots show replications (*n* = 3) within samples. Lines connect the means. TOC—total organic carbon, TN—total nitrogen.

The initial difference between composted substrates was assessed by the differences in the dynamics of chemical and enzyme parameters it feels redundant. The decomposition process was evidenced by a decrease in respiration and the loss of substrate mass, carbon, cellulose, and hemicellulose content, with differing dynamics for two substrates (Figure 2A). During the oat straw decomposition, the microbial activity (accessed by the carbon dioxide emission from substrates) was the highest in the early and middle phases and rapidly declined toward the end, indicating the completion of the decomposition of the most available organic compounds. The leaf litter rates in the early and late phases were not significantly different, which may be linked to the initial smaller content of available nutrients. Along with the microbial activity, the process of decomposition for both substrates was marked by the mass loss of the decomposing substrate, which coincides with the loss of organic compounds. For the oat straw, 72.65% of the mass was decomposed by the late phase of the experiment, whereas the leaf mass loss by the late phase amounted to only 47.55%. In both substrates, the hemicellulose undergoes decomposition by the early phase, while the cellulose content in the substrate remains practically unchanged. A significant decrease in cellulose content was detected in the middle phase for the oat straw and the late phase for the leaf litter. This also could be linked to a higher content of humic compounds in straw compared to leaf compost.

The total carbon content decreased gradually in both substrates as they decomposed, while the total nitrogen content shifted in waves: an increase followed by a decrease, indicating succession in the microbial communities. The leaf compost at the beginning of composting had a pH value slightly more acidic than the straw compost due to differences in substrate acidity. During the composting process, an increase in pH was observed for leaf litter and a slight decrease for straw.

The dynamics of the enzyme groups of interest were shown to shift according to the stage of degradation and substrate (Figure 2B). Both oat straw and leaf litter were characterized by a decrease in the activity of peroxidase, invertase, amylase, and protease by the late stages of decomposition. Agreeing with the data of chemical analysis, higher enzyme activity was shown in the decomposition of oat straw. Straw compost showed higher activity of catalases, endocellulases, exocellulases, peroxidases, and proteases. Invertase was more active in leaf litter. Polyphenol oxidase in straw compost was the most active in the middle phase, while in leaf litter, it was in the last. Thus, significant differences in the rate and efficiency were observed in the decomposition of cellulose-containing substrates differing in nutrient availability.

### 3.2 The composts’ taxonomic composition

**Figure 3:**
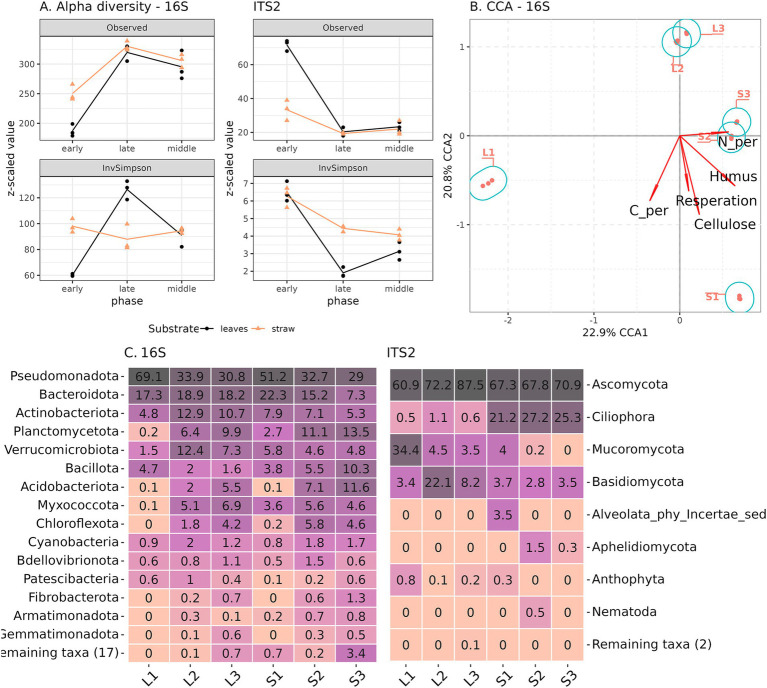
Prokaryotic and eukaryotic composition of composts accessed by 16S rRNA gene and ITS2 fragment Illumina sequencing. **(A)** Alpha-diversity, **(B)** CCA for 16S rRNA, and **(C)** Phylum composition (relative abundance). S1–S3—samples from straw compost, L1—L3—from leaf litter.

We attempted to link the previously discussed differences in the chemical composition of composts with the dynamics of their taxonomic composition. After filtering unidentified phylum-level ASVs and organelle reads, a total of 2,244 phylotypes were identified for the 16S rRNA gene and 250 for the ITS2 fragment libraries. We observed a different pattern in taxonomic dynamics for prokaryotes and eukaryotes of microbial communities during plant residue decomposition. For prokaryotes, there was an increase in the richness (number of observed ASV) of both composting substrates from early to late stages (Figure 3A). Evenness (inverted Simpson index) increased in leaf litter compost and decreased in straw compost. Both richness and evenness of the eukaryotic component were characterized by a decline in diversity from the early to the late decomposition stages. The richness values were different in the beginning (in leaf litter higher), but at the late stage, they were equally low. Initially, the evenness values were similar, but in straw, at the later stages, values were higher. Thus, the prokaryotic and eukaryotic components of the decomposing community were characterized by reverse dynamics—an increase in the richness of the former and a decrease in the latter.

The beta-diversity of the microbiomes showed that the prokaryotic communities from the early phases of both composts were the most distinct from each other, while the middle and late phases for each substrate were similar (Figure 3B). The same was observed for eukaryotic communities, except that all phases of straw compost were quite close in composition (Supplementary Figure S1). According to the CCA, carbon content coincided with the dynamics of the decomposition, while respiration, pH, TN, cellulose, and humus content were associated with the substrate differences.

Since the experiment used a soil-based microbial inoculum for the initiation of the decomposition, the taxonomic composition of the composts’ prokaryotic community was very diverse at the phylum level. The early phase consisted mostly of Pseudomonadota and Bacteroidota phyla, but during the composting process, their relative representation decreased in favor of other phyla (Acidobacteriota, Planctomycetota, Myxococcota, Chloroflexota, and Cyanobacteria) (Figure 2C). In the straw compost, Bacillota increased their abundance during decomposition, while in leaf litter, they decreased. On the genus level in the early phase, both composts were dominated by *Flavobacterium*, *Pseudomonas*, *Pseudoxanthomonas*, and *Chitinophaga* (Supplementary Figure S2). In addition, in straw compost, we detected *Devosia*, *Luteimonas*, and *Sphingobacterium*; in leaf, we detected *Paenibacillus*, *Novosphingobium*, *Allorhizobium*, and *Galbitalea*. In the later phases, both composts were inhabited by *Ohtaekwangia*, *Acidibacter*, and *Steroidobacter*. Straw was characteristic of *Bacillus*, *Sphaerisporangium*, and *Clostridium*; leaf was characteristic of *Terrimonas*, *Verrucomicrobium*, and *Bauldia*. The diverse eukaryotic community of the early phases of decomposition was gradually replaced by *Ovatospora* (Ascomycota) in both composts, which was especially characteristic of leaf litter (Figure 3C; Supplementary Figure S2). The late phase of the straw compost became inhabited by ciliates *Gastrostyla* and *Gonostomum*. Potentially pathogenic fungi *Alternaria*, *Pyrenophora*, *and Lichtheimia* were present at the early phases of decomposition but not detected in the mature composts.

### 3.3 The composts’ metagenome analysis and functional composition

**Figure 4:**
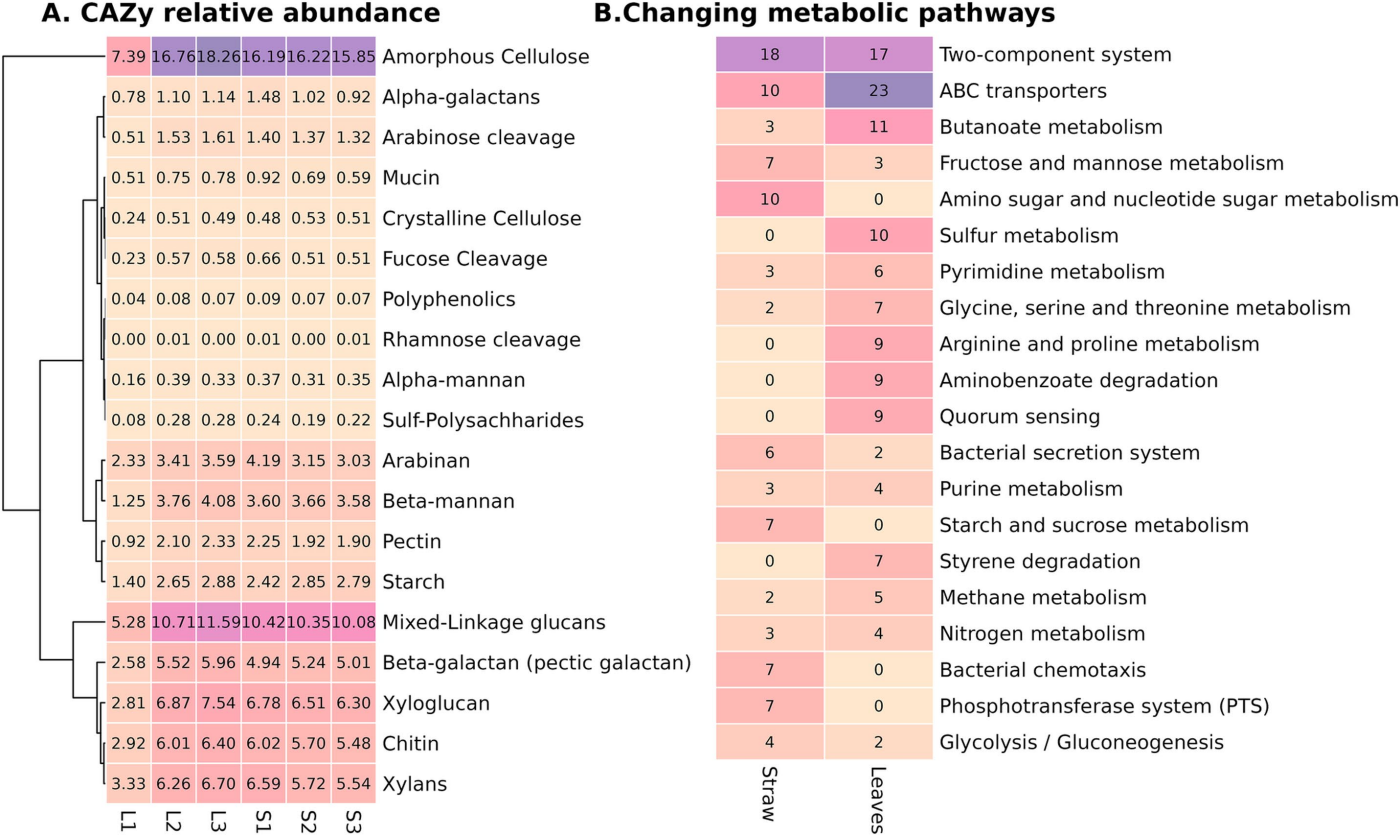
Metagenome characteristics of the contrasting composts: **(A)** Relative abundance (in percentiles from the ORFs from the metagenome) of CAZY genes united in DRAM categories in the six samples, **(B)** Number of KO (KEGG orthology) categories in the 20 most abundant metabolic pathways, significantly different between compost types.

After nanopore sequencing, six metagenomes (three for straw and three for leaf litter) with a total read length of 190.4 Gb were obtained. The technical information on the quality of reads after basecalling and the quality of the resulting assemblies is presented in the Supplementary Table S2. To characterize functional differences between composts, we compared full metagenome annotations, specifically CAZy genes. The composition of CAZy genes, linked to the polysaccharide’s utilization, did not show significant variability either between substrate types or phases of decomposition, except for the early phase of leaf compost, which had the lowest values across all samples and DRAM categories (Figure 4A). The most abundant CAZy gene categories were amorphous cellulose, mixed-linkage glucans, xylans, xyloglucan, and chitin.

Following assembly and subsequent annotation, 14,691 KO categories were identified in the metagenomes. After filtration by the representation, 5,793 KO categories were used for the differential analysis to assess significant differences between substrates, treating all phases from one substrate as replicates. As a result, 263 KO categories were significantly increased in straw and 258 KO categories were significantly increased in leaf litter (Supplementary Figure S3). The most represented category was the two-component system; each substrate was more abundant in specific KOs from this category (Figure 4B). Differences in the two-component system suggest that the leaf compost microbiome may experience catabolite repression, low nitrogen availability, and oxygen limitation (Supplementary Figure S4). The second most abundant category was ABC transporters, and the leaf compost had more than twice the amount of KO compared to straw. Notably, leaf litter compost was more abundant in taurine and alkanesulfonate uptake, which coincides with the fact that it was also more abundant in KO from sulfur metabolism. Straw compost was enriched in the metabolism of simpler compounds (starch and sucrose, amino sugar, fructose, and mannose), while leaf litter was more abundant in KO from pathways of aromatic compounds degradation (xylene, benzoate, aminobenzoate, furfural, dioxin, and hydroxy phthalate) (Figure 4B; Supplementary Table S3). The metagenome of the straw compost had more KO, indicating the presence of microbial interaction—bacterial chemotaxis, antibiotic resistance, and flagellar assembly. Both composts were enriched in nitrogen metabolism, but straw compost had a pronounced denitrification pathway. Diversification in secretion-type systems was also noted—while straw compost was more prevalent in types II, III, and VI, leaf litter was more prevalent in types I and IV. Straw was enriched in biotin and zinc uptake.

Consistent with the Illumina data, a considerable amount of contigs in the metagenomic assembly of early phases of composting was annotated as belonging to the Pseudomonadota phylum, which decreased in later phases (Supplementary Figure S5). Yet, even in the early phases, the proportion of genes associated with cellulose degradation in this phylum in the metagenomes was lower in both substrates compared to other phyla, e.g., Actinobacteriota, Planctomycetota, Bacteroidota, and Bacillota (Supplementary Figure S5). The most characteristic was the difference between the earliest phase and the rest for both substrates. Oat straw was characterized by a high proportion of endocellulases in Bacillota and Verrucomicrobiota representatives. Furthermore, the representation of these gene groups in the minor communities only continues to increase. For the leaf litter community, lower diversity of these genes (cellulase, catalase, and amylase) in minor phyla was observed, with higher representation in representatives of Actinobacteriota and Bacteroidota. Therefore, during the decomposition, we observed an increase in the taxonomic diversity of the functional genes.

### 3.4 MAGs and PULs from the compost’s metagenomes

After we did not detect significant differences in CAZy composition between the composts, we shifted the analysis from the level of full metagenomes to metagenome-assembled genomes (MAGs) and polysaccharide utilization loci (PULs). A total of 57 high and 240 medium-quality MAGs were obtained from the metagenomes. Of these, 254 contained clusters of carbohydrate-active genes (CGCs). In turn, PULs were identified in 188 genomes, of which 135 had clusters capable of cleaving the *β*-1,4 bond between glucose residues, which is characteristic of cellulose and hemicellulose. We compared the CGC/PUL ratio in MAGs from different phyla (Supplementary Figure S6) to elucidate possible distortions in the data we obtained, connected with the underrepresentation of non-Bacteroidota phyla in the dbCAN-PUL. All ratios strived toward 50%, except MAGs from Acidobacteriota and Verrucomicrobiota, although it may be caused by a small number of CGCs in them. The completeness of the genome assemblies did not affect these results. Thus, PUL quantities should adequately describe cellulolytic potential in the observed MAGs.

The highest number of PULs among all composts was observed in the representatives of Bacteroidota, Pseudomonadota, Actinomycetota, Bacillota, and Chloroflexota (Supplementary Table S4). The most numerous target substrates of their PULs were pectin, xylan, and arabinan. Pseudomonadota were also abundant in PULs for target substrates, not directly connected with cellulose degradation, such as capsule polysaccharide synthesis, glycosaminoglycan, and starch.

**Figure 5:**
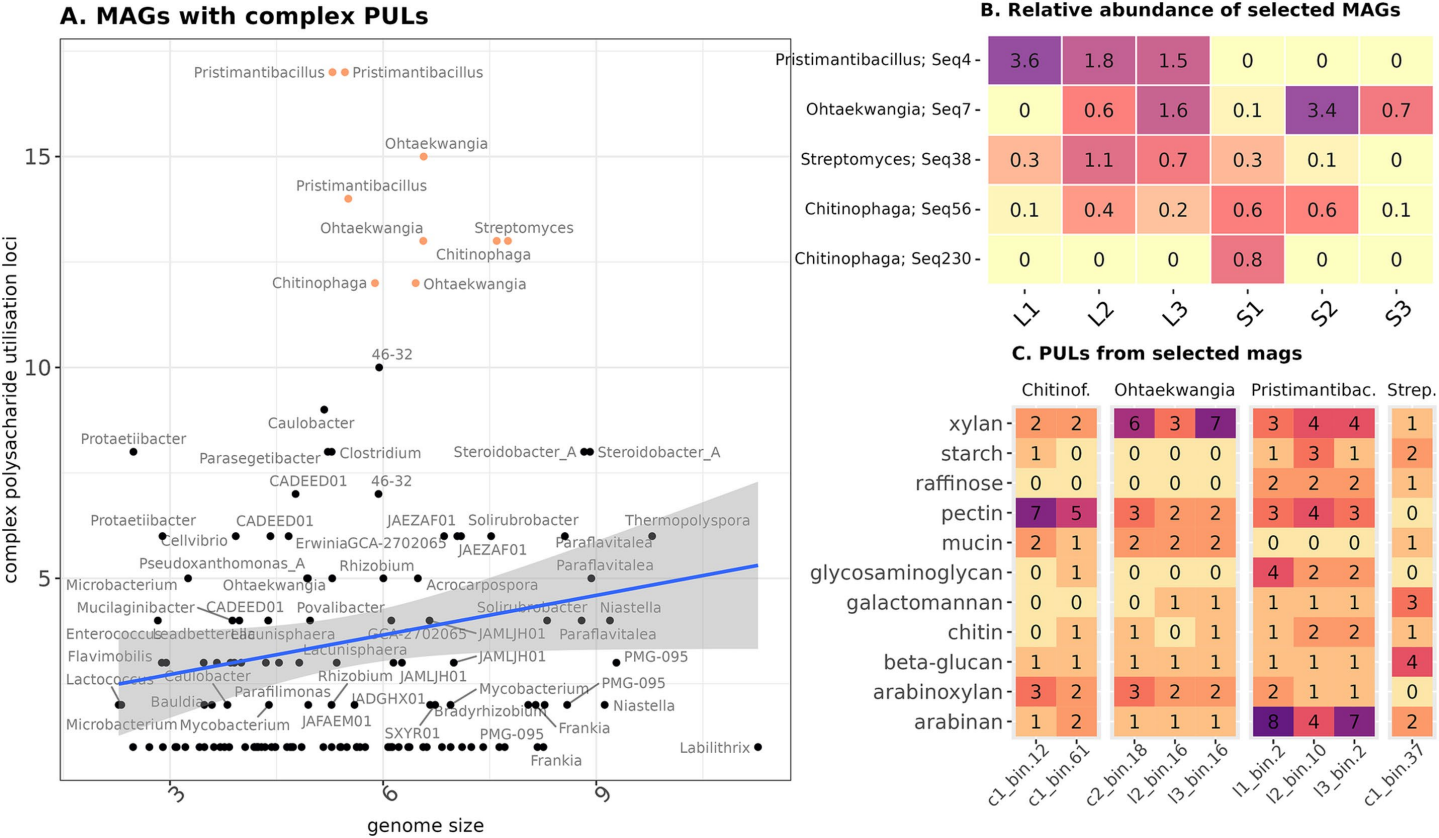
Characteristics of the most potential cellulolytic MAGs. **(A)** Ratio of PULs linked with lignocellulose degradation to the genome size. The group selected for further analysis is highlighted in color. **(B)** Presence of the chosen MAGs in the composts according to the Illumina data. **(C)** PUL substrates found in the selected MAGs. Genus names are given according to the GTDB annotation and match those on the left plot.

The data obtained allowed us to identify the most potentially active cellulolytic organisms from the microbial communities of both composts. These were nine MAGs, containing an abnormally high proportion of PULs relative to their genome size (Figure 5A). They attributed to Bacteroidota (*Chitinophaga* in straw and *Ohtaekwangia* in straw and leaf litter), Actinobacteriota (*Streptomyces* in straw), and Bacillota (*Pristimantibacillus* in leaf litter). There were other bacteria, mostly belonging to the Pseudomonadota, which contained high amounts of PUL, but their PULs used pectin as a target substrate or were involved in cell capsid degradation. We found a match for these MAGs with five phylotypes from 16S rRNA gene amplicon sequencing data (Figure 5B). Two bacilli genomes belonged to the same phylotype, and one representative of *Chitinophaga* aligned to a minor phylotype, probably due to nanopore sequencing error. The remaining phylotypes belonged to the major component of the community. *Bacillus* and *Streptomyces* were characteristic of the early stages of leaf decomposition, and *Chitinophaga* was most represented in the early stages of straw decomposition. The representative of *Ohtaekwangia* did not show substrate specificity in relative representation and appeared only at the late stages of decomposition.

The identified PUL target substrates varied between genera of MAGs. *Chitinophaga* was the most abundant in PULs specific for pectin, *Ohtaekwangia*—xylan, *Pristimantibacillus*—arabinan, and *Streptomyces*—beta-glucan (Figure 5C, Supplementary Table S5). Other target substrates, for which PULs were present, included arabinoxylan, mucin, glycosaminoglycan, chitin, and starch. “PULs of simpler polysaccharide compounds from MAGs assembled in this study shared high similarity with the reference ones, e.g., in *Chitiniphaga* – glucomannan, Streptomyces – beta-glucan, *Ohtaekwangia,* and *Pristimantibacillus* – pectin (Supplementary Figure S7). At the same time, PULs for xylans were mostly different in organization from the reference ones.

## 4 Discussion

In the present study, cellulolytic communities of different decomposition stages of oat straw and leaf litter were selected as the objective of the investigation. The use of a compost based on the soil of Chernevaya taiga soil as an inoculum was motivated by the fact that, although a significant number of active cellulolytic organisms have been described in the literature, majority of them have been isolated from rumen and intestinal microbiomes (Chassard et al., 2010), and soil microorganisms are extremely underrepresented in the databases. The advantage of soil microbiota over rumen is in the potentially higher adaptation to the decomposition of a wider range of plant residue types (Bao et al., 2021). Understanding which part of the microbial community has the greatest potential for lignocellulosic complex decomposition may allow a shift from microbial preparations based on individual strains to more effective ones based on microbial consortia (Bradáčová et al., 2019; Liu et al., 2023). This is specifically important because soil communities are highly diverse and relatively stable, which complicates the integration of microbial preparations based on single strains (Kaminsky et al., 2019; Ke et al., 2021). The soil of Chernevaya taiga, formed in unique environmental and geochemical conditions, is specifically promising in finding such microbiota.

We used two contrasting lignocellulosic substrates—oat straw and leaf litter. The dynamics of chemical characteristics and differences in the activity of the enzymes in both composts may indicate that oat straw in the experiment was more saturated with water-soluble hydrocarbons and proteins relative to leaf litter, while the latter was more recalcitrant due to higher content of resilient materials, not covered by our analyses. In addition, while organic carbon content was similar, nitrogen content was higher in straw, so the C/N ratio, which affects the activity of microbiota (Azis et al., 2023; Guo et al., 2018), was more favorable in straw than in leaf litter. The lower C/N ratio could also explain the release of mineral nitrogen in the middle phase of oat straw decomposition (Janssen, 1996), which additionally facilitated the endo-and exo-cellulolytic activity of the microbiota. The presence of bacterivorous ciliates in straw compost may serve as further evidence of the faster turnover of the organic material (Bick and Müller, 1973). Consistent with this, in our experiment, straw turned out to be a more accessible substrate and decomposed much faster than leaf litter.

As stated above, while both straw and leaf litter are cellulosic substrates, their resistance to decomposition is different due to chemical characteristics. This establishes conditions for the mobilization of different parts of the initial microbial soil community into the enrichment culture. We observed this effect in the taxonomic structure of the compost microbial communities, which shifted significantly between the types of substrates. However, both composts shared similar patterns of microbiome development. Our choice of longer periods of sample incubation had a significant effect on the outcome: we detected long-term shifts in microbial and functional composition, with an increase in bacterial diversity and a decrease in fungi, coinciding with one of our previous experiments (Kimeklis et al., 2023). The observations of dynamics at the taxonomic level are consistent with the earlier findings, specifically the prevalence of Pseudomonadota during the initial stages of straw colonization from soil and the diversification of the community in the later phases (X. Wang et al., 2022; Kimeklis et al., 2023). Genera detected by Illumina sequencing in both types of composts—*Pseudomonas*, *Bacillus*, *Flavobacterium, Sphingobacterium, and Novosphingobium*—were reported multiple times to contain potentially active cellulolytic species (Lednická et al., 2000; Kim and Yu, 2020; Cheng et al., 2019; Goswami et al., 2022). Cellulose-degrading strains from *Pseudoxanthomonas suwonensis* (Hou et al., 2015) and *Luteimonas* (Lin et al., 2020) were isolated from the soil. Hence, while both composts were diversified in major microbiota, majority of them comprised potential decomposers. Despite this, on the taxonomy level of analysis, we cannot determine which members of the community had this predisposition, especially considering that the microbiomes of the middle and late phases in both composts were very close, but the enzymatic activity between these phases shifted very prominently. Moreover, earlier findings showed that only a minority of a bacterial community in the compost may be associated with lignocellulosic substrate degradation (Sun et al., 2023). Therefore, we used metagenomic data to look for potential active decomposers in both communities.

Despite the strong variation in the activity of cellulolytic-related enzymes between the phases of decomposition and substrate types, we did not detect significant shifts in CAZy categories between the six analyzed metagenomes. Consistent with this, no enrichment of any CAZy families was shown between metagenomes of microbial communities isolated from different substrates (Gladkov et al., 2022). The activity of catalytic enzymes decreased by the late phases of decomposition, while the relative content of genes associated with lignocellulose decomposition remains stable. While we did not dwell on this matter more closely, we can speculate that this regulation is performed on the level of gene expression, which, in turn, is regulated at the cellular level by regulatory systems (e.g., two-component systems) responding to the substrate representation in the environment. This assumption is evidenced by the overrepresentation of diverse KO groups from two-component metabolic pathways between compost types. Another important observation is that although Pseudomonadota contributed a lot to the metagenome on the level of all annotated genes, their contribution to the genes associated with cellulose decomposition was smaller. Therefore, we can assume that members of minor phyla were potentially more involved in cellulose-degrading activity than members of Pseudomonadota (Supplementary Figure S5). This effect is particularly evident in the late stages of decomposition and is substrate-dependent. A similar effect was shown previously, as we detected that a significant part of microbial taxonomic diversity at the later phases of straw colonization does not participate in cellulose degradation because it does not have genes for the corresponding enzymes in its genomes, while these groups of microorganisms can make a serious contribution to the community by shifting the representation of effective cellulolytic microorganisms (Kimeklis et al., 2023).

To identify the most probable active decomposers of the microbial communities of the composts, we searched for MAGs with an abnormally high content of PULs relative to their genome size. The previous study on lignocellulose biomass decomposition indicated that the most promising degraders, according to the GH gene content, belong to Bacteroidota and Bacillota (Huang et al., 2023). Consistent with their findings, according to Illumina sequencing, Bacteroidota and Bacillota phyla were abundant in both composts, and out of nine MAGs, which we selected as promising active decomposers according to our criteria, five belonged to the first and three to the latter. Notably, Actinobacteriota was also reported to play an important role in the lignocellulose degradation (C. Wang et al., 2016), but only one potentially cellulolytic MAG from this phylum was assembled, which may be linked to its genome size (Seshadri et al., 2022). All these MAGs were the representatives of both minor and major microbiota, indicating the involvement of the diverse ecological groups in the process of decomposition.

These data could not be checked for significance, but the PULs specificity seems to be connected to the MAG taxonomic attribution, not the phase of decomposition from which it was detected. Since we observed the taxonomic differentiation of potentially active MAGs between composts, we can assume that PUL specificity is also highly linked with the substrate type. Interestingly, Huang et al. (2023) used CGCs instead of validated PULs because majority of their data were not indexed in dbCAN-PUL. We managed to work on the PUL level, but majority of their target substrates were compounds, accompanying cellulose fibrils. Along with that, the PULs, specific for cellulose degradation, were absent in almost all MAGs, selected as potentially active degraders. This may be a consequence of the fact that the PUL database mostly covers compounds, usually found in the gastrointestinal tract, which is the most frequent object for studying polysaccharide degradation. Thus, our study demonstrates that soil communities decomposing lignocellulose can also be a valuable source for the identification of novel PULs.

All the above shows that the design of effective lignocellulosic communities requires an integrated approach, which is not limited only to the analysis of individual enzyme groups or individual taxa. It is important to understand the principles of formation and functioning of the cellulolytic microbial consortium to apply this knowledge to the formulation of highly effective microbial preparations.

## 5 Conclusion

The composts based on two types of lignocellulosic biomass were studied with a comprehensive approach using the combination of analyses of enzyme activities, Illumina Miseq read-based sequencing of the 16S rRNA gene and ITS2 fragment amplicon libraries, and metagenome analysis using Oxford Nanopore MinION long-read technology to search for promising cellulolytic prokaryotes. The complementarity and convergence of the two sequencing methods in the context of soil metagenomics were demonstrated. We have shown that in a long-term experiment on the decomposition of plant residues, despite the use of an inoculum prepared from one soil, succession of different microorganisms occurs in different composts. The rates of dynamics of chemical parameters and changes in taxonomic composition do not coincide, which indicates an incomplete correspondence between the functional potential and the taxonomic composition of the community. The same is confirmed by the fact that in the studied composts, the dynamics of enzymatic activity associated with the decomposition of plant residues did not coincide with the dynamics of the CAZy genes. However, the analysis showed that functional differences between composts were revealed not at the level of individual genes but at the level of their organization into clusters. Moreover, the analysis of MAGs of potential cellulolytic microorganisms showed that some of them are substrate-specific and are major representatives of the microbial community.

## Data Availability

The datasets presented in this study can be found in online repositories. The names of the repository/repositories and accession number(s) can be found in the article/Supplementary material.
