# Supplementary material for: Metagenomic insights into the development of microbial communities of straw and leaf composts
Source: Front Microbiol. 2025 Jan 22;15:1485353. doi: 10.3389/fmicb.2024.1485353 (PMC11794307; doi:10.3389/fmicb.2024.1485353)
Supplement: Supplementary file 2 [file Data_Sheet_1.docx]

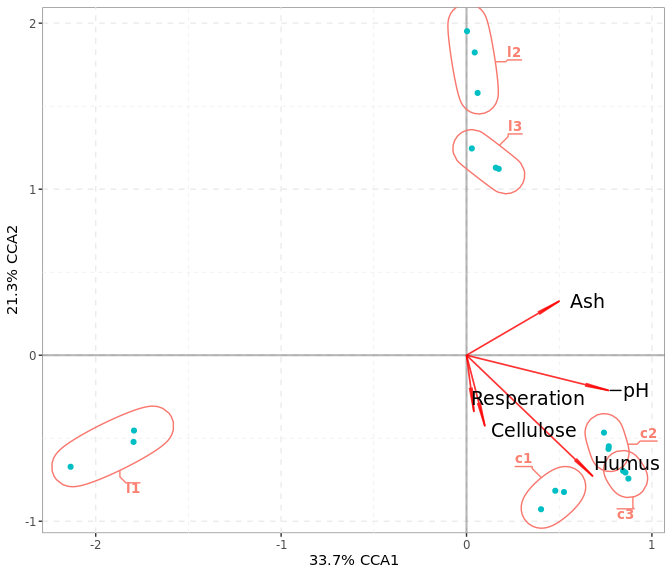


Figure S1. CCA for ITS data


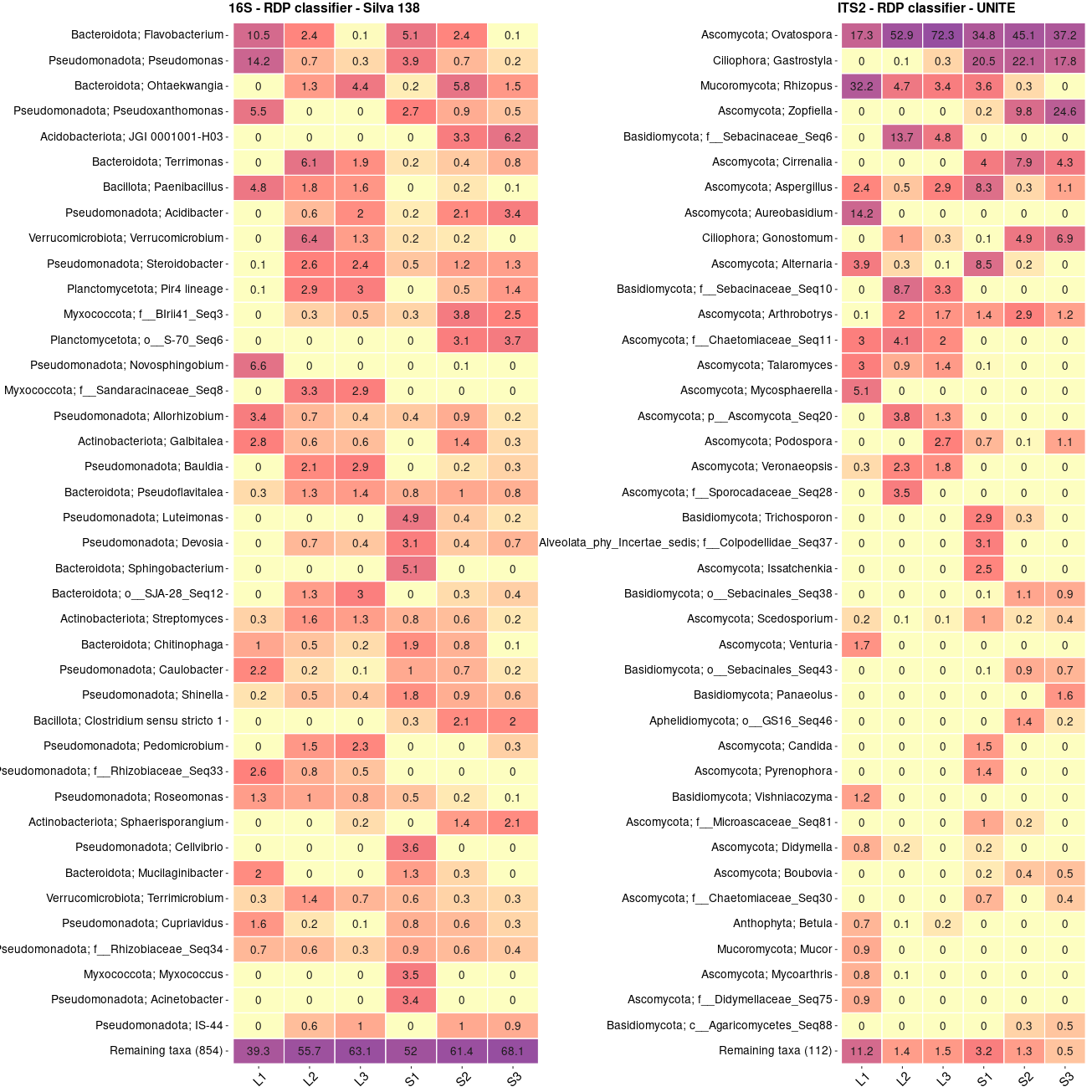


Figure S2. Heatmap of composts composition on the genus level. Left – bacterial taxonomy based on 16S rRNA gene annotation by Silva 138, Right - eukaryotic taxonomy based on ITS2 fragment annotation by Unite.


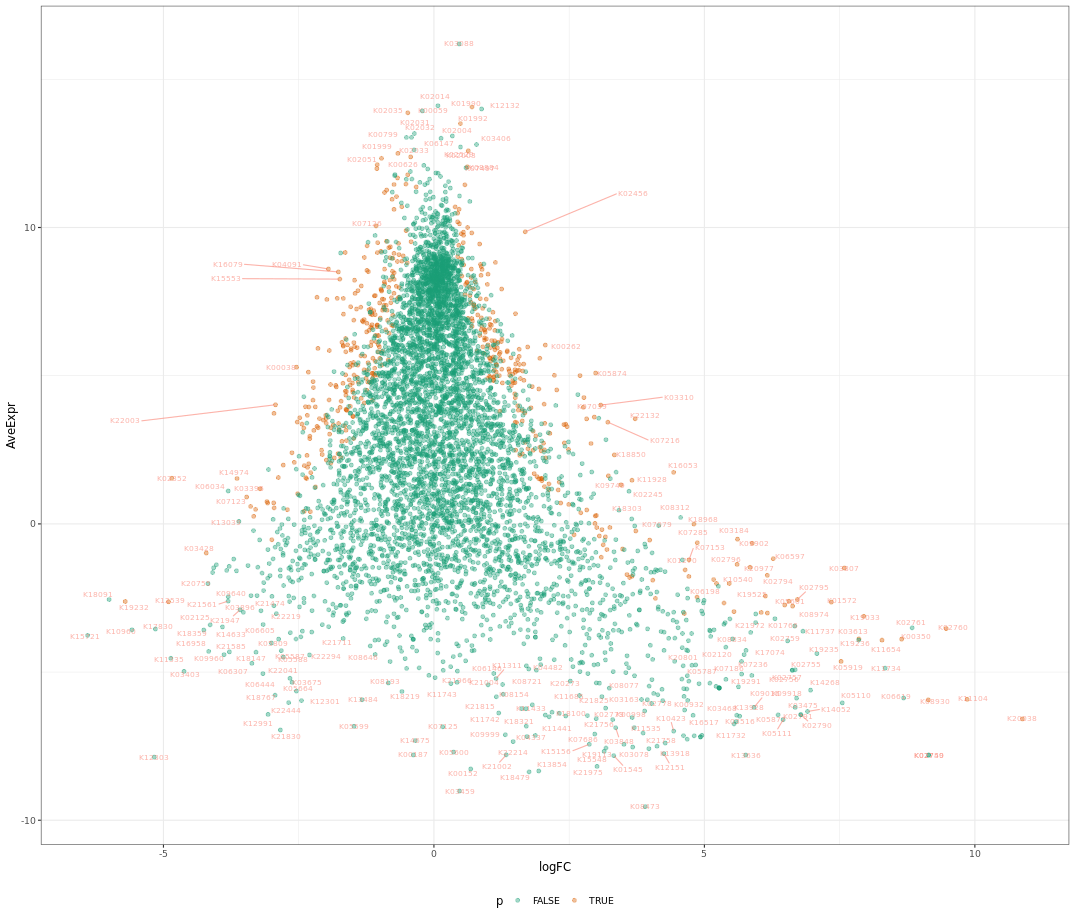


Figure S3. Differential abundance of KO categories between composts on contrasting substrates.


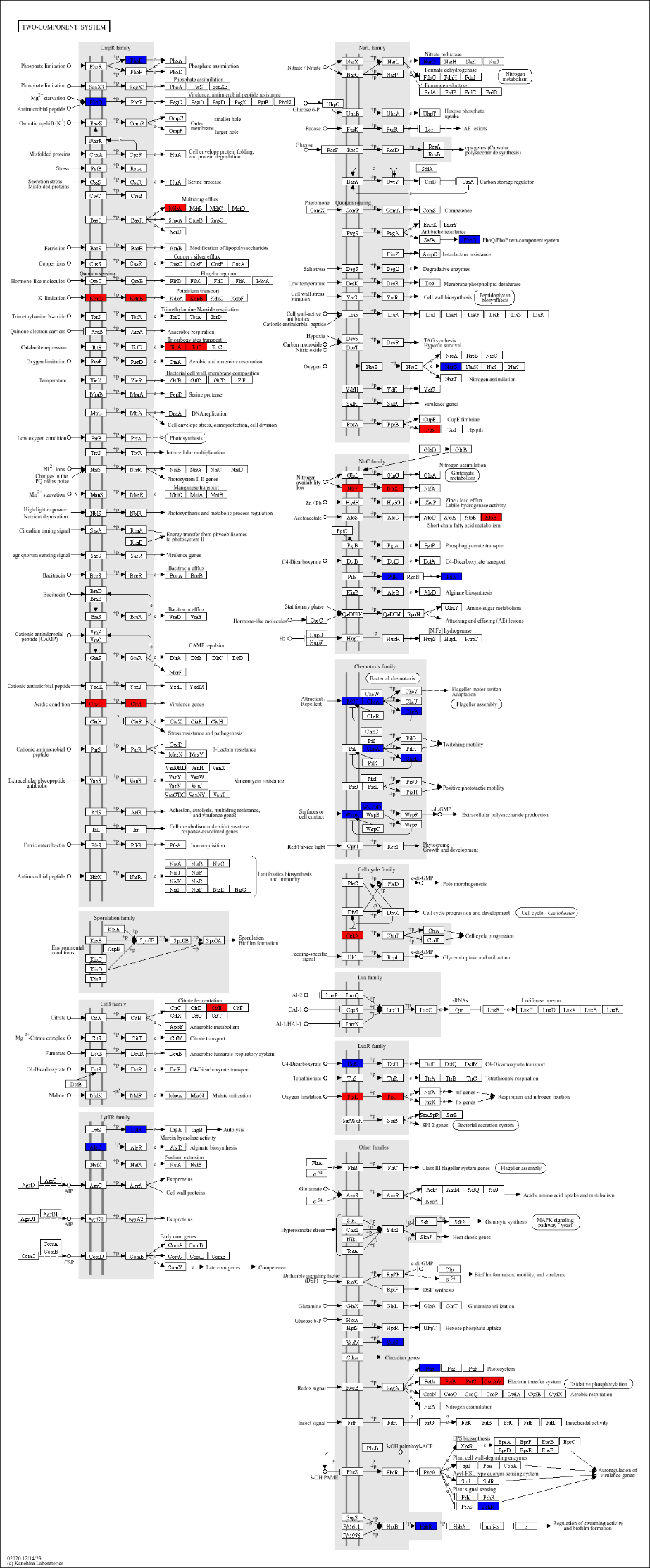


Figure S4. Two-component system metabolic pathway. Blue – KO, enriched in straw, red – in leaves.


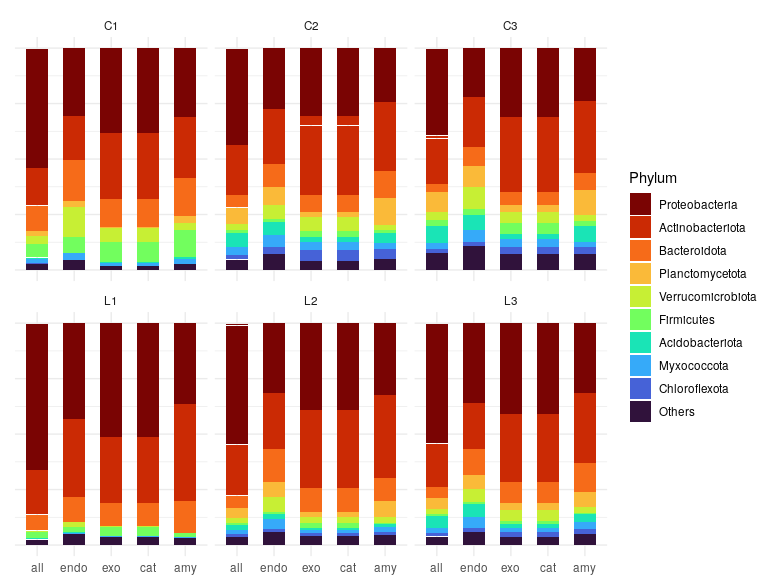


Figure S5. Taxonomic attribution of functional genes at different stages of decomposition. All – all ORFs in the metagenomes. Endo, exo, cat, amy – cellulolytic-related KO (endocellulases, exocellulases, catalases, amylases), coding for enzymes, used in the analysis. C1, C2, C3 – three phases of straw decomposition; L1, L2, L3 - three phases of leaf litter decomposition.


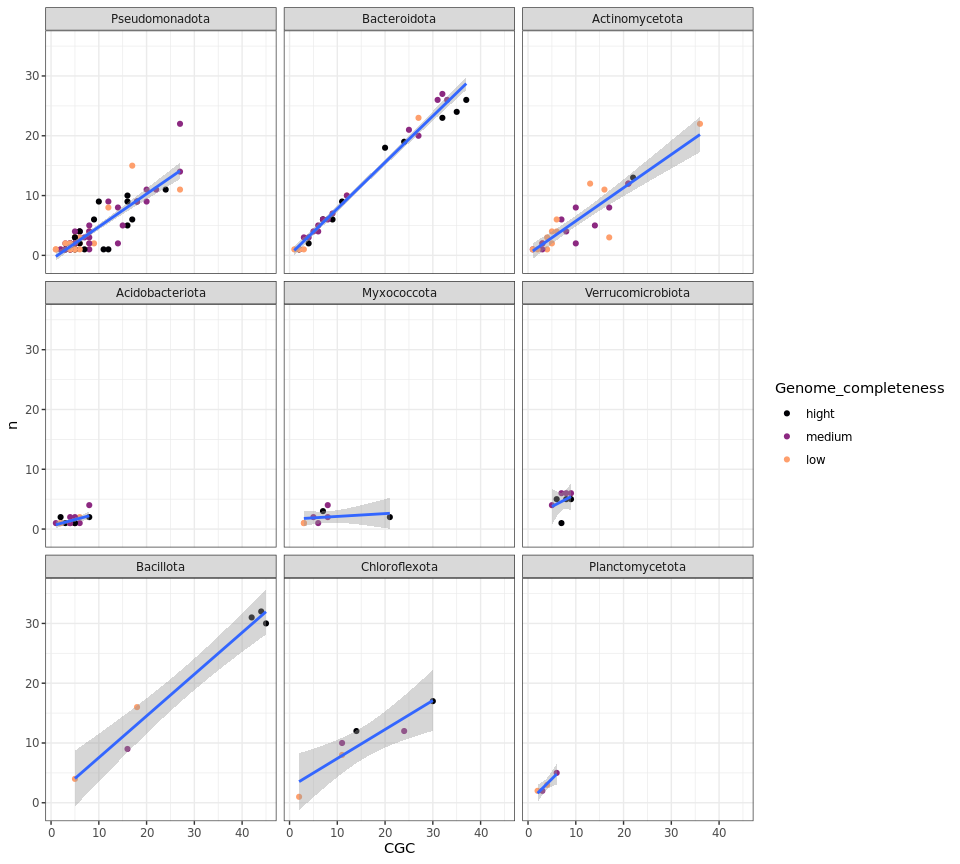


Figure S6. Dependence of the number of PULs (n) on the number of CGCs in a phyla-dependent manner. The color indicates the completeness of genome assembly (high > 90%, low <75%, and medium).

| Chitinophaga | Pectin  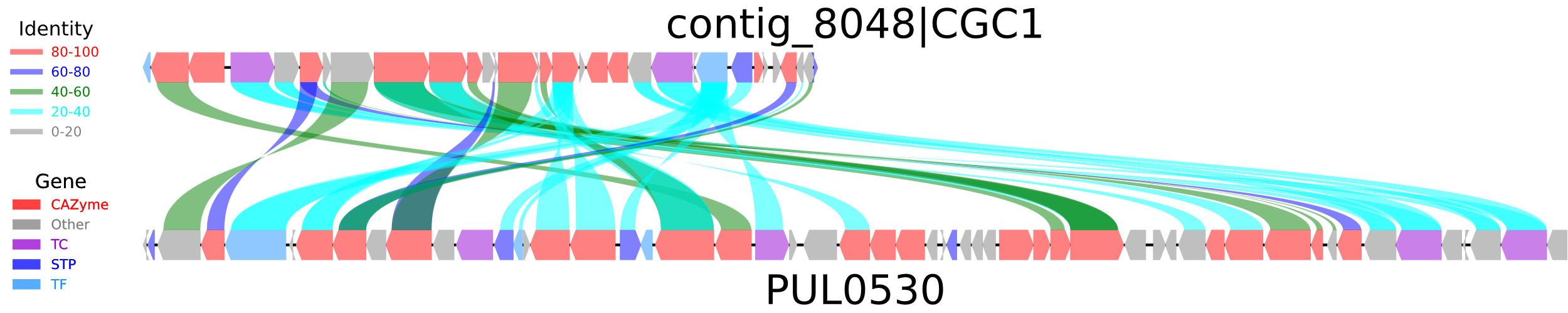  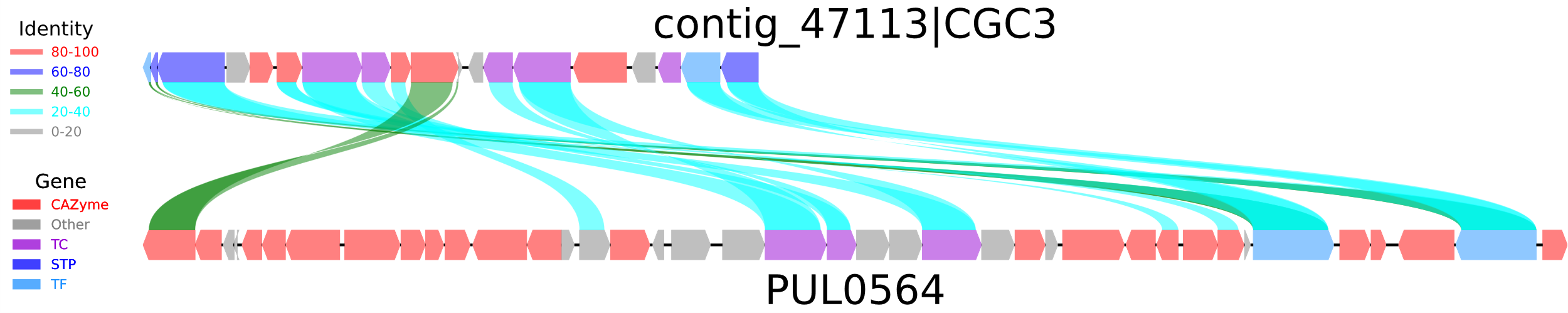  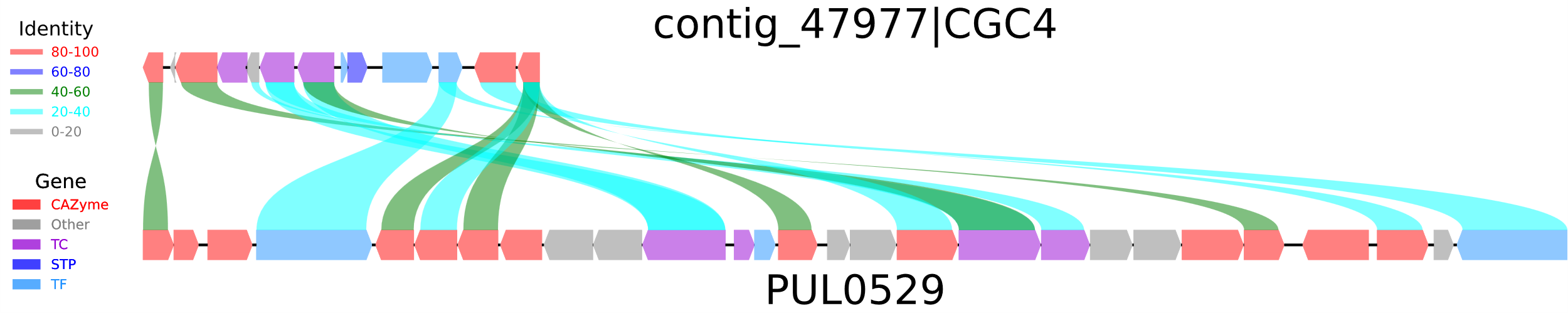 |
| --- | --- |
|  | Xylan  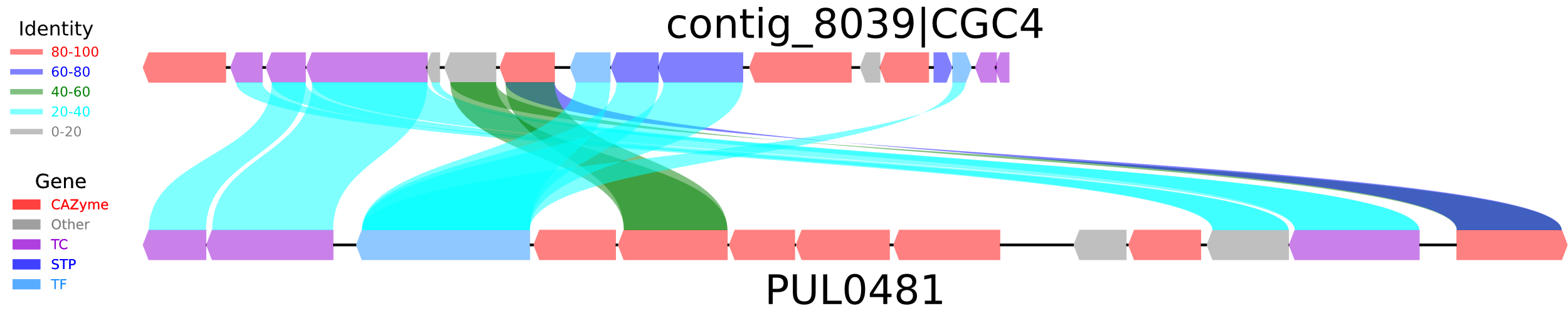  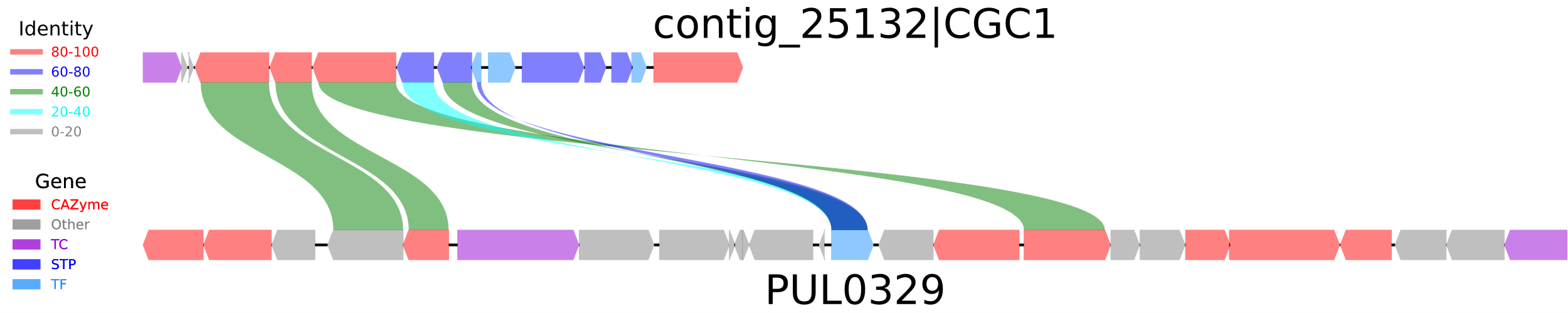 |
|  | Arabinoxylan  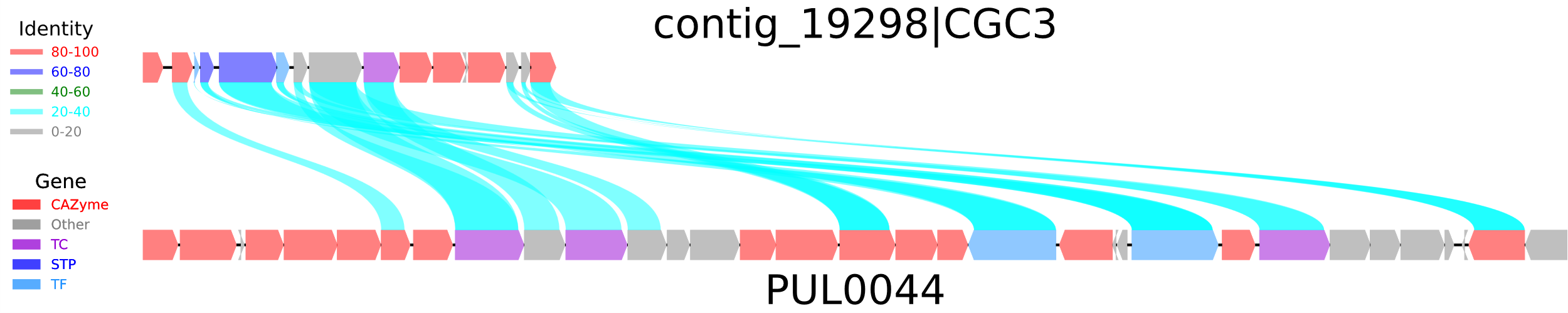 |
|  | Glucomannan  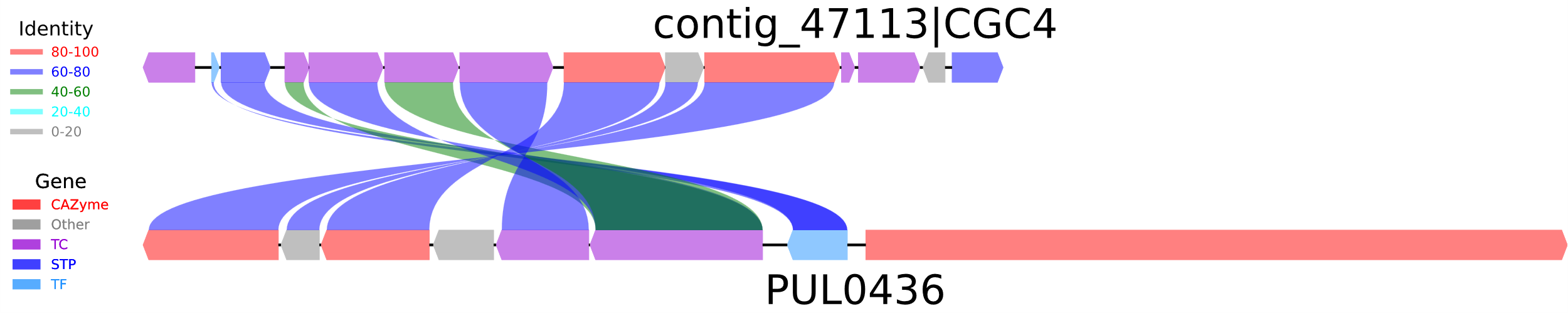  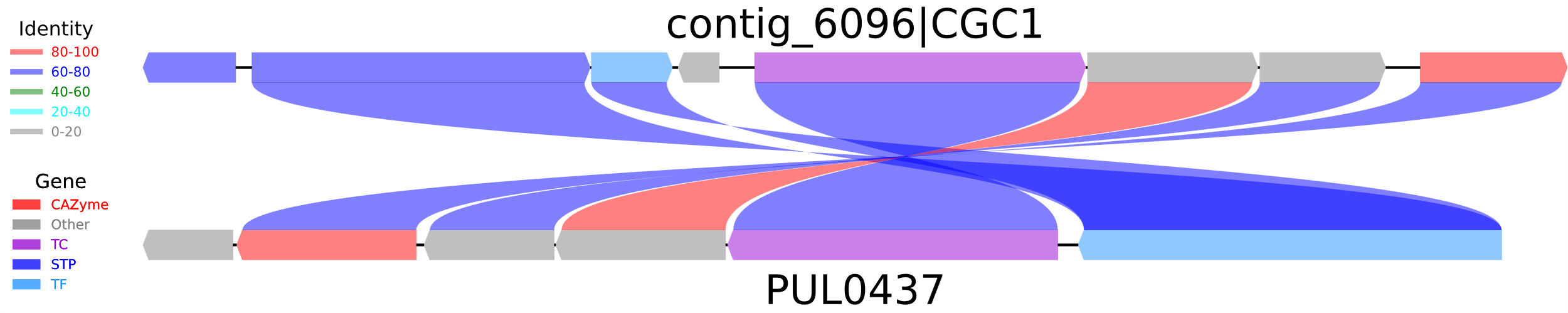  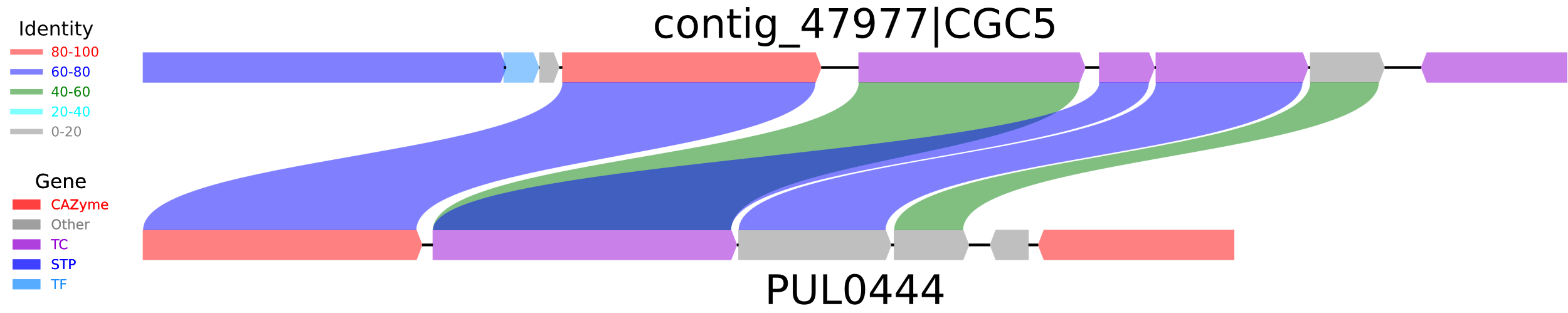 |
|  | Beta-glucan  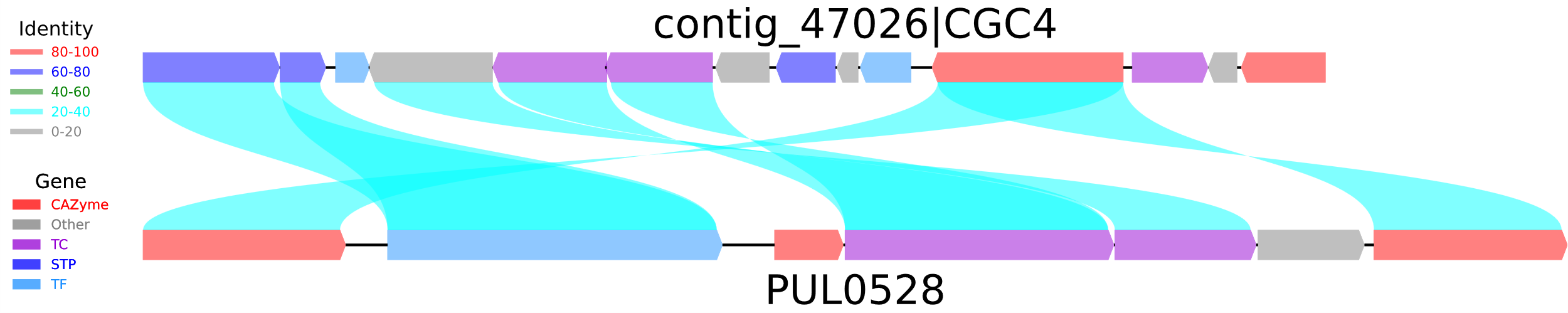 |
| Streptomyces | Beta-glucan  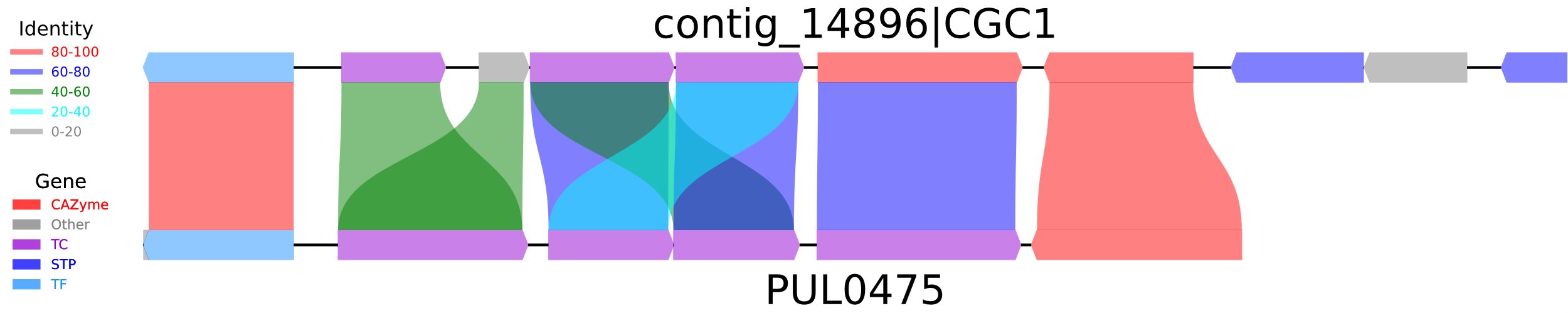  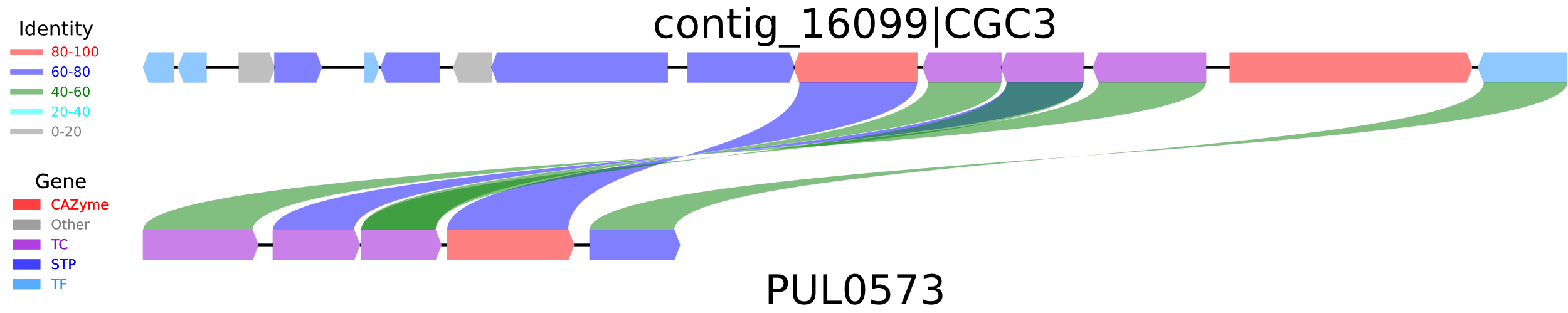  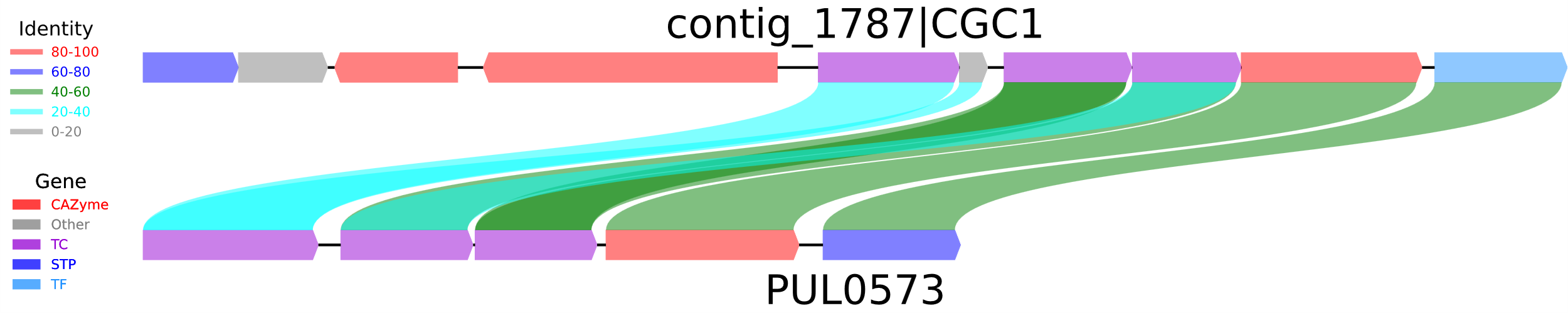 |
| Ohtaekwangia | Xylan  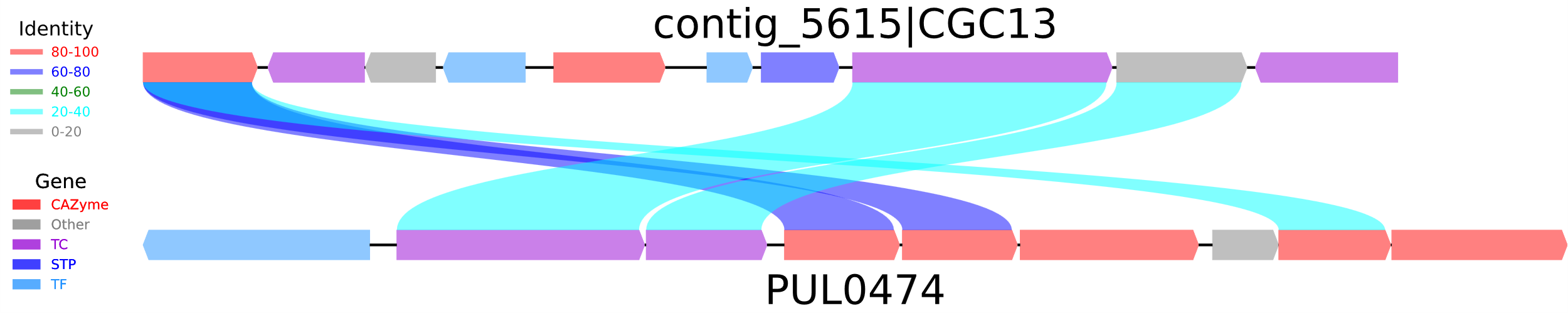  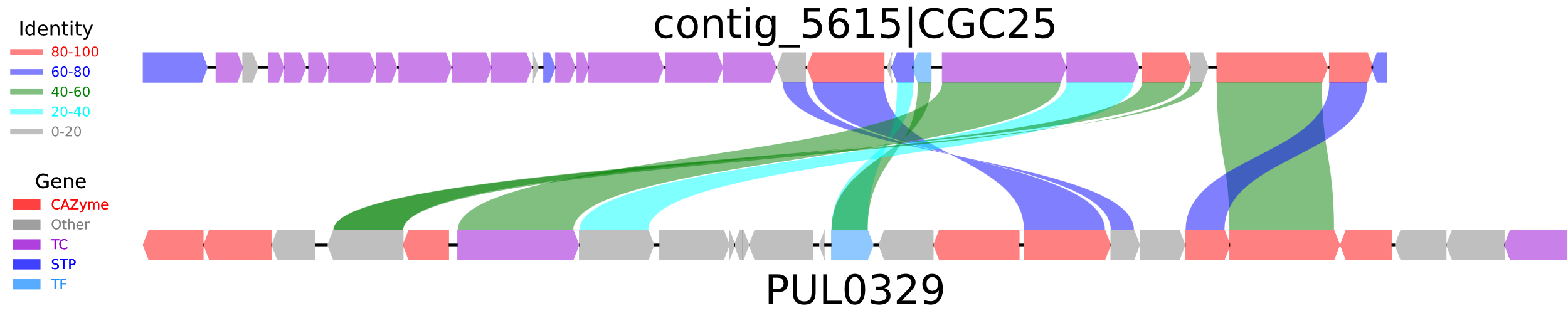  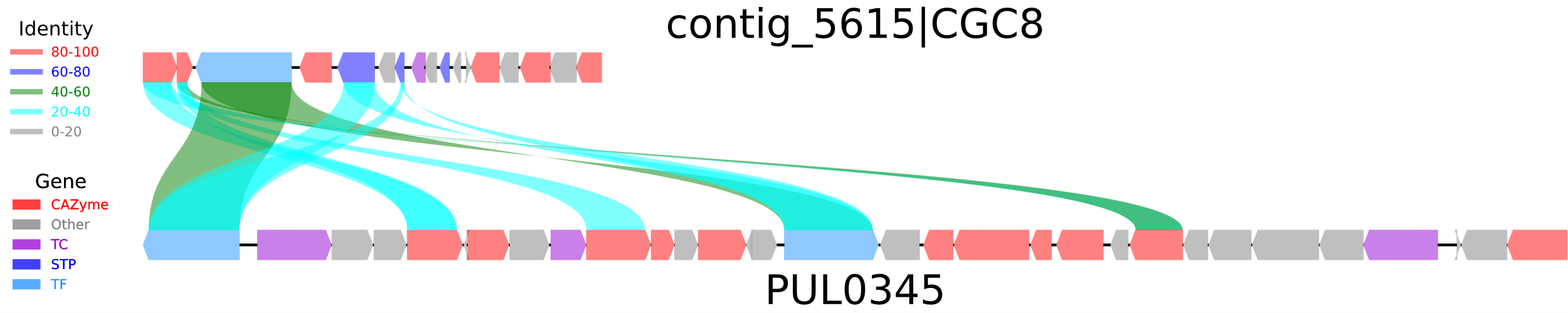  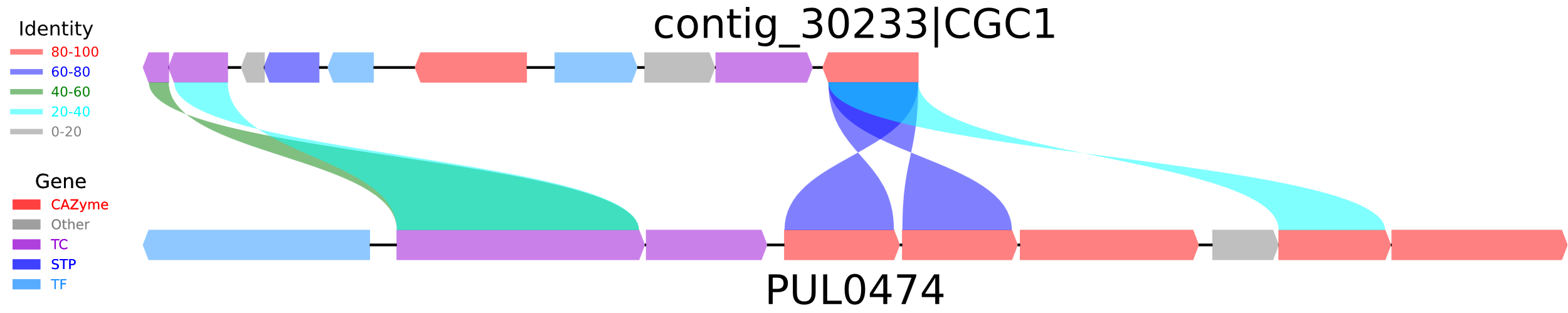  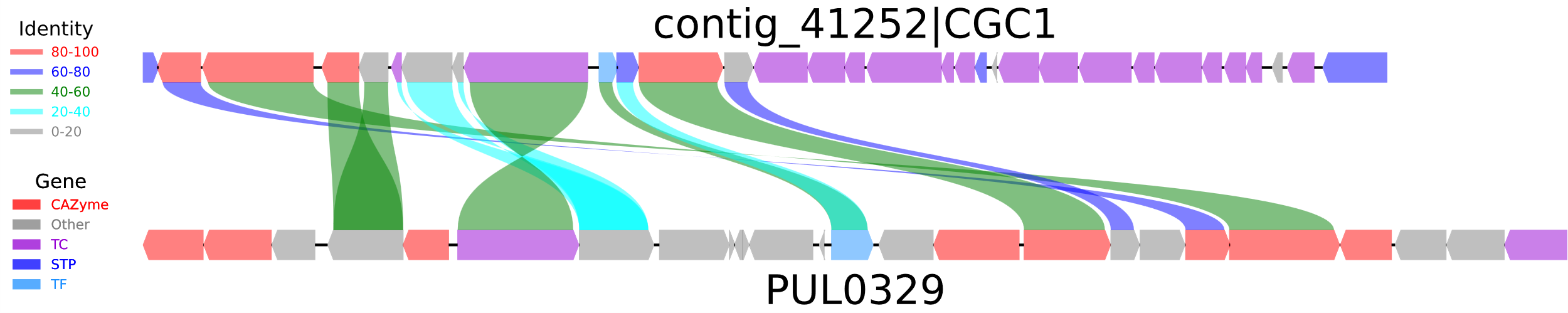  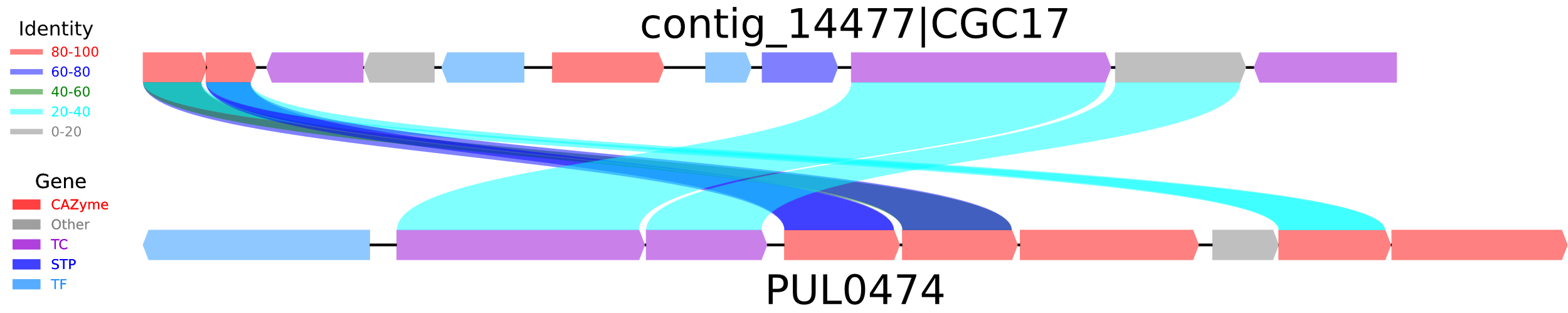  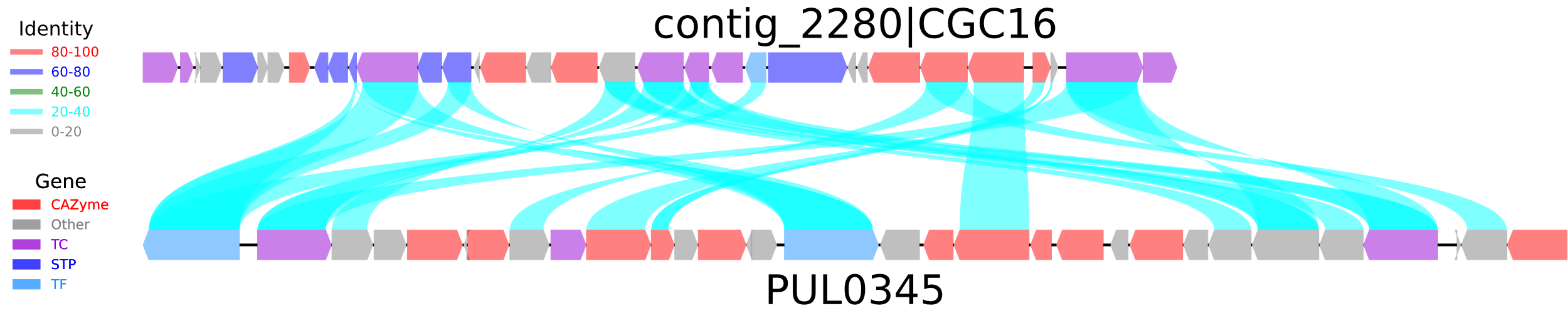  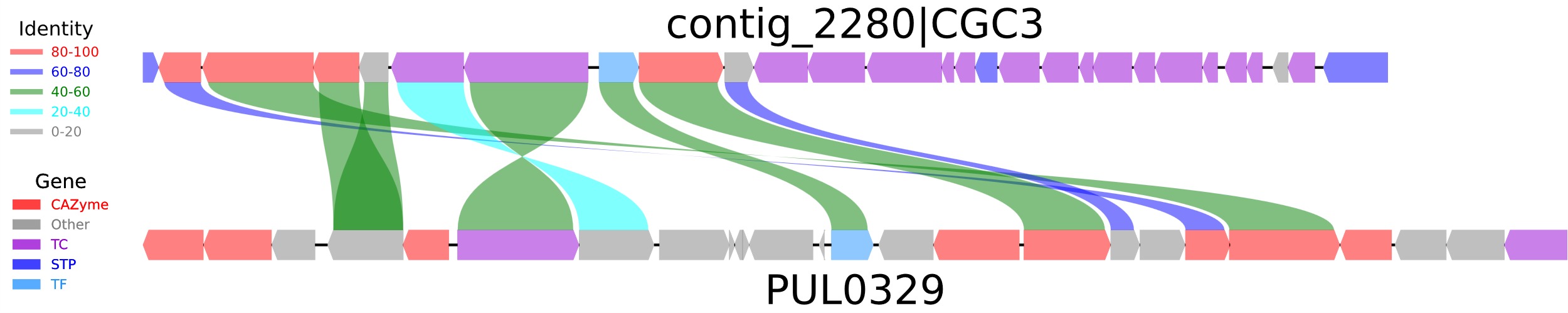 |
|  | Arabinoxylan  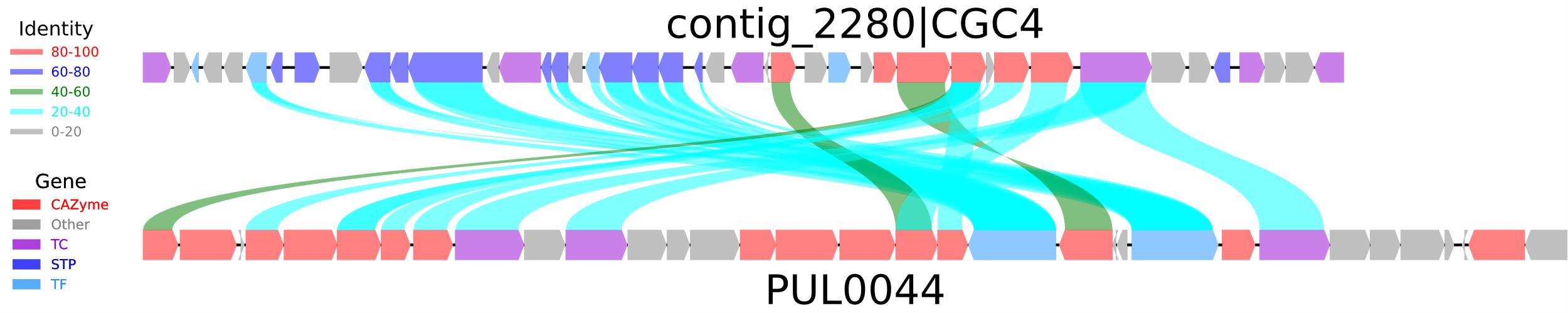 |
|  | Pectin  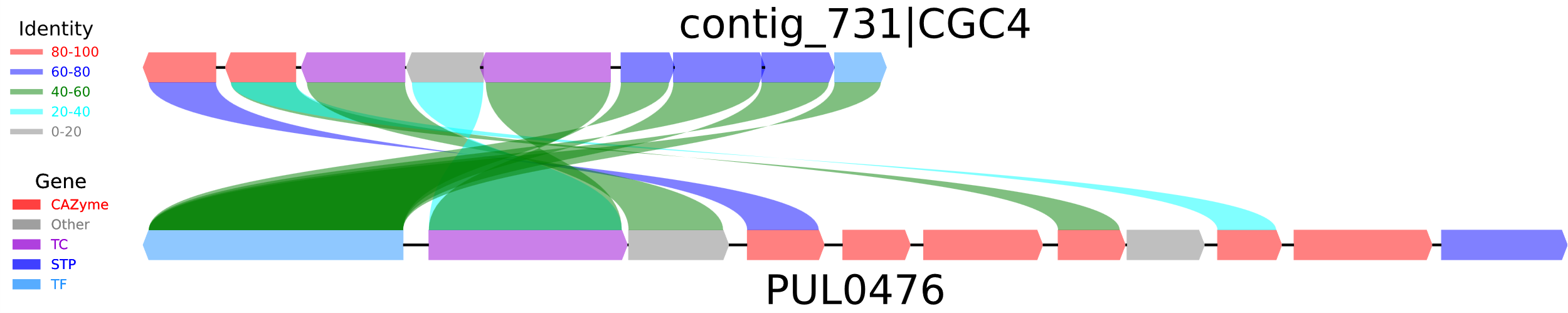  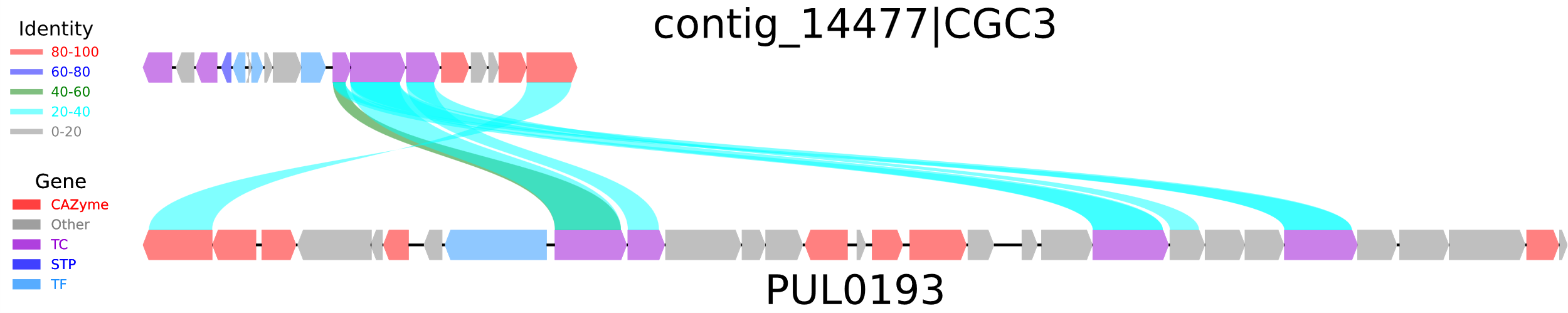  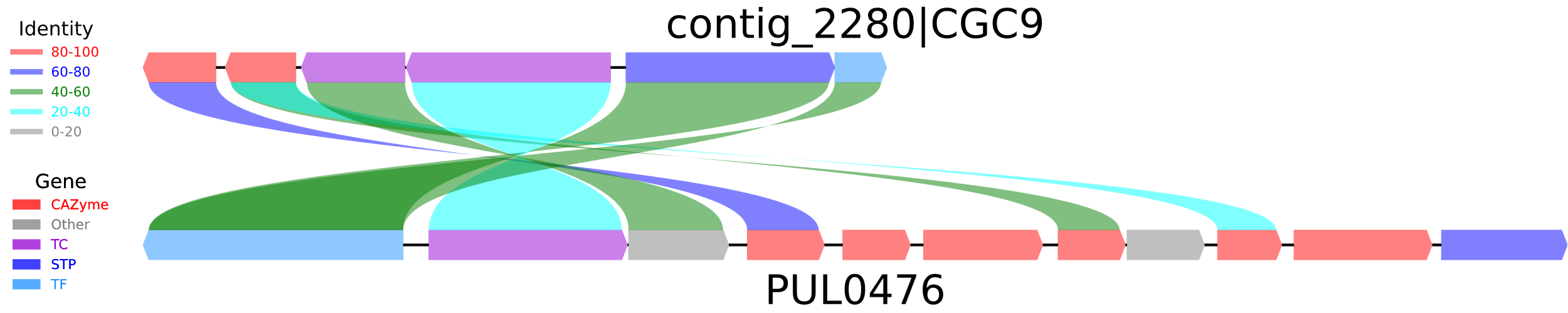 |
|  | Glucomannan  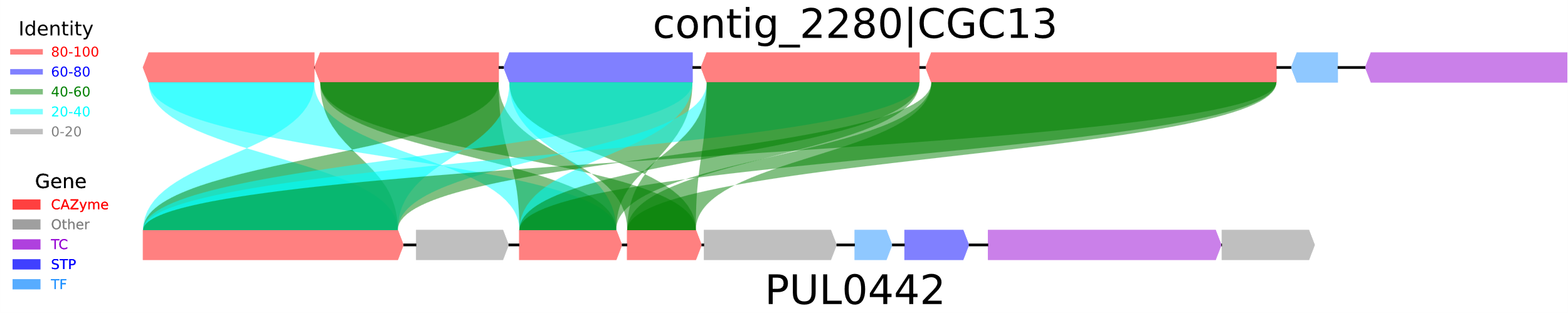 |
|  | Carrageenan  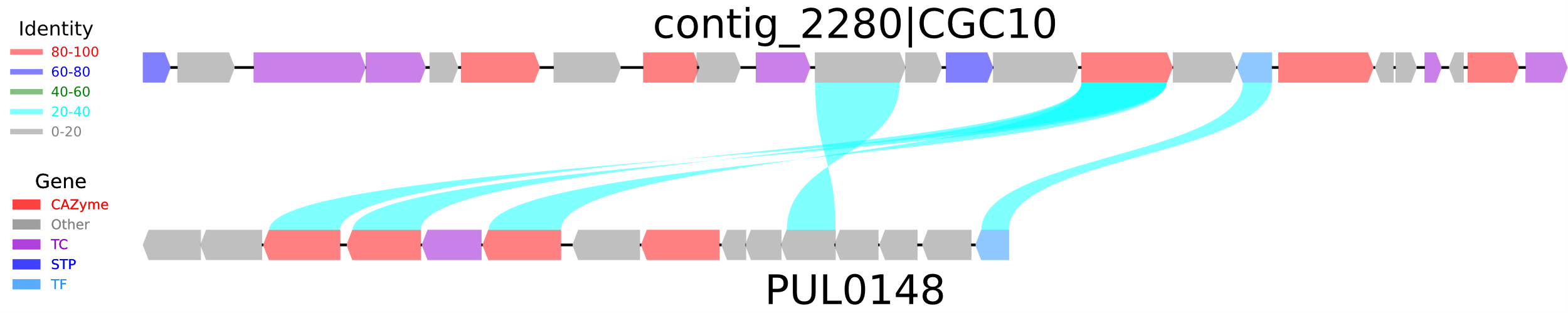 |
| Pristimantibacillus | Betaglucan  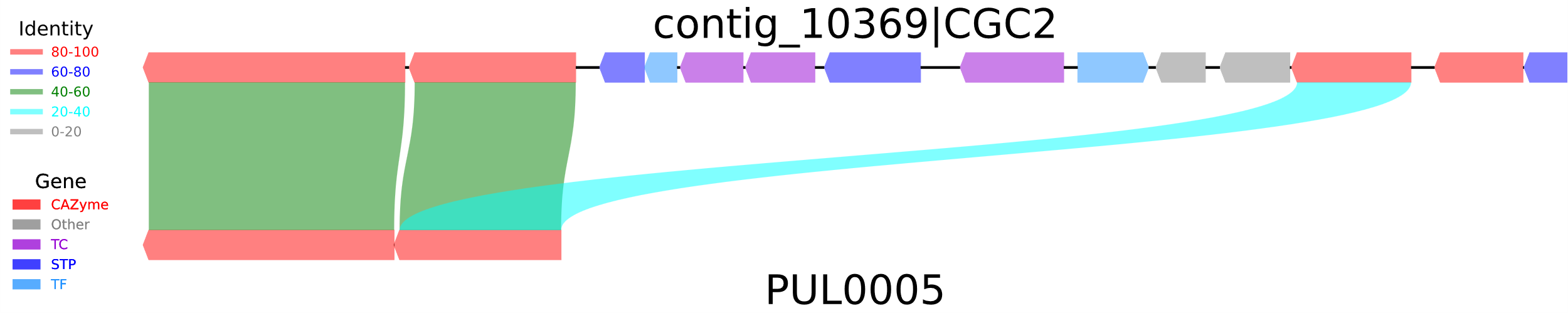  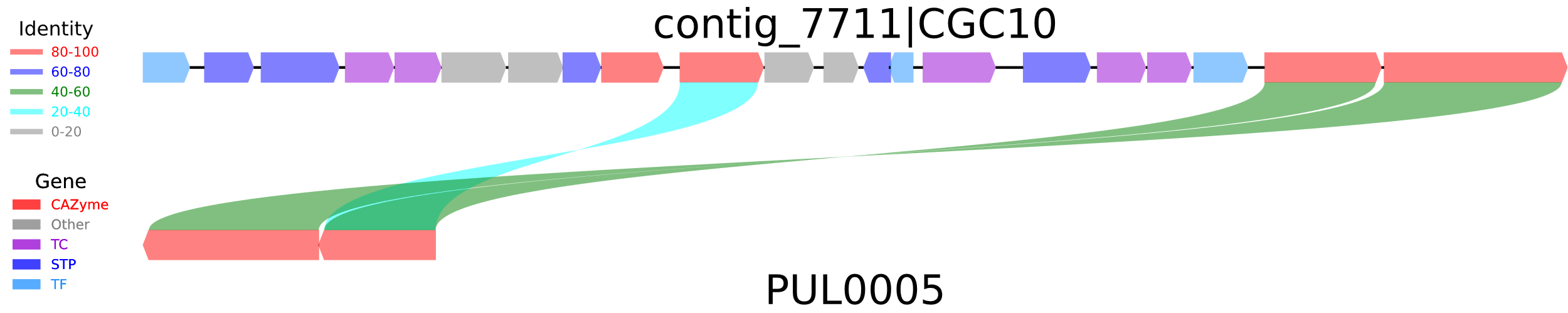  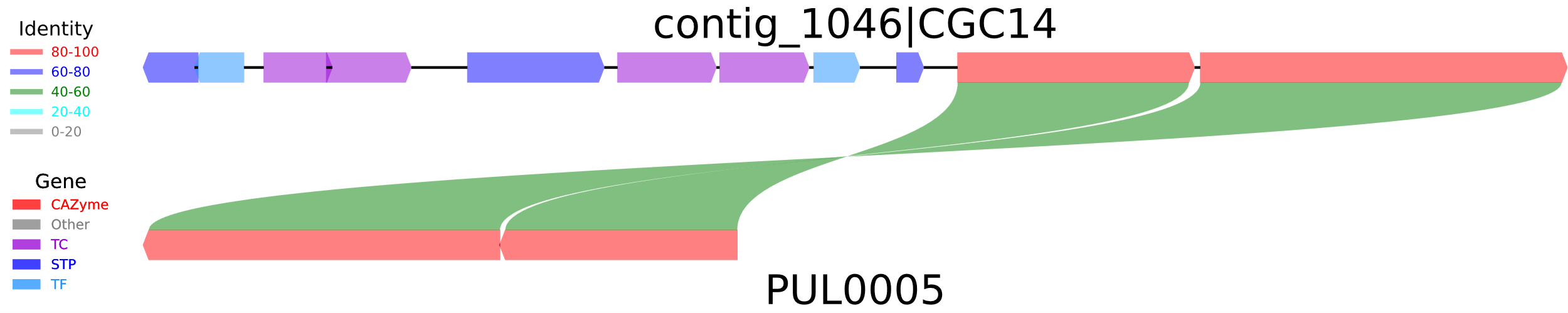 |
|  | Arabinogalactan  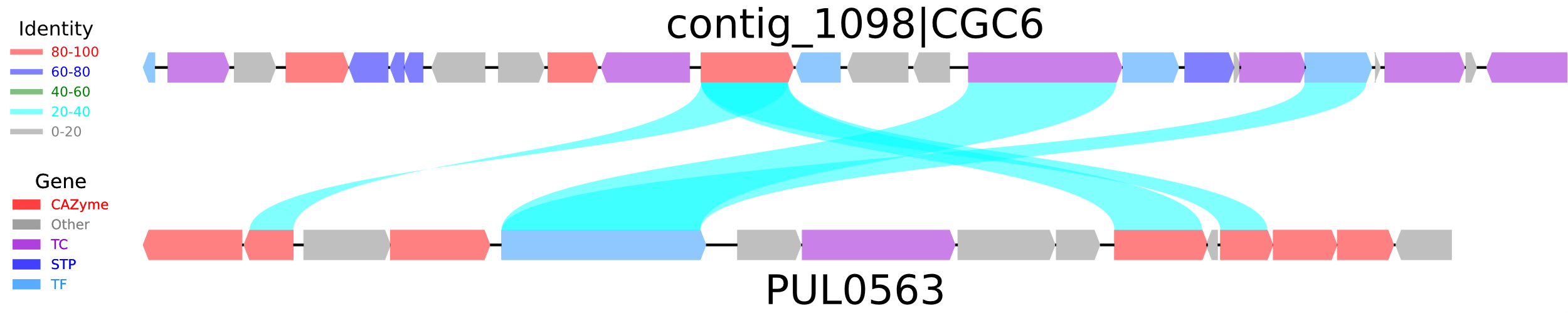 |
|  | Pectin  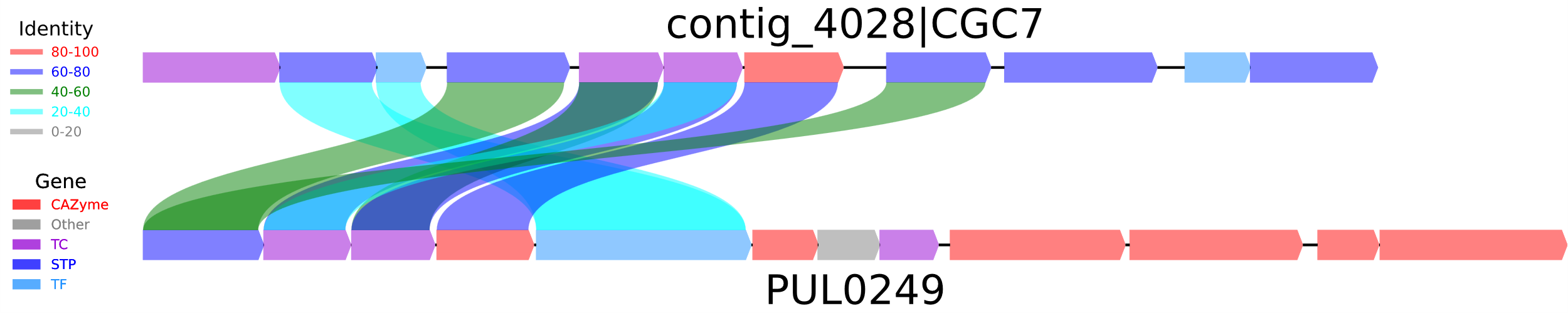  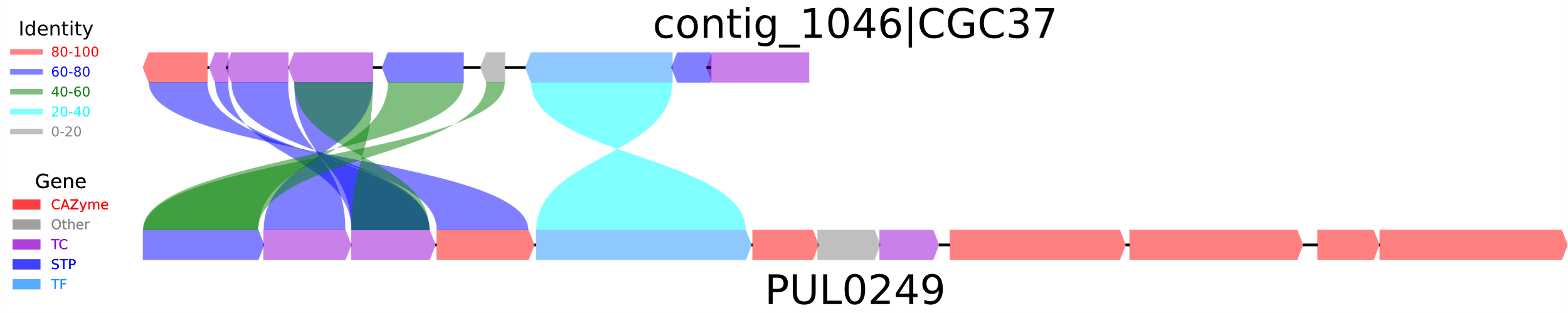 |
|  | Xylan  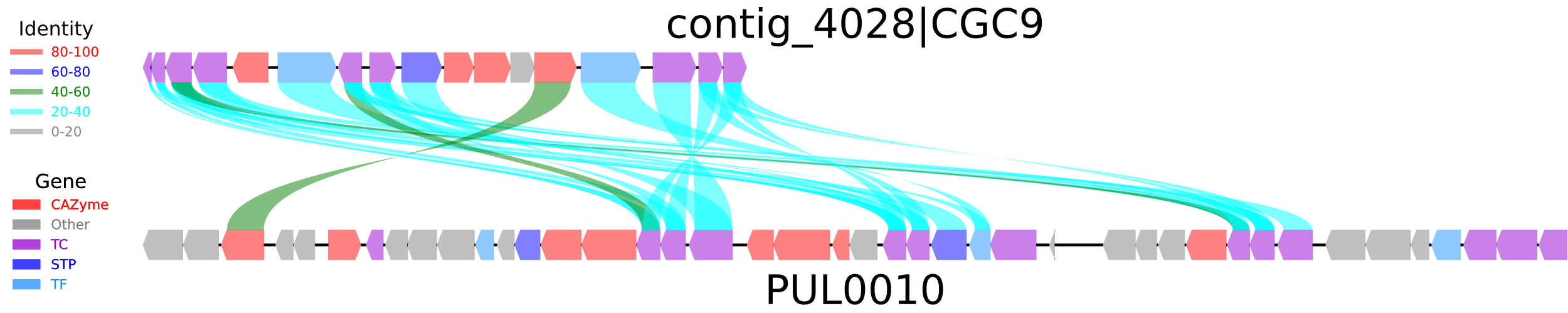  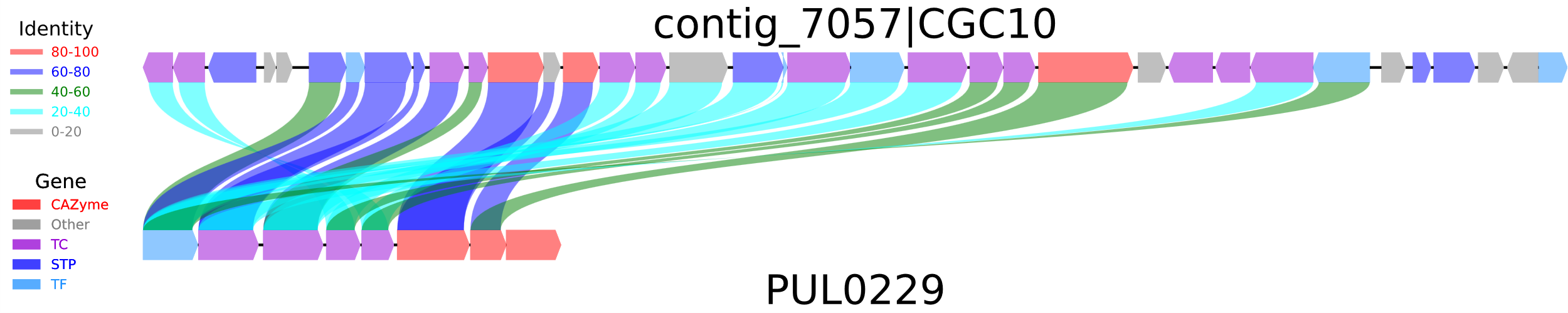  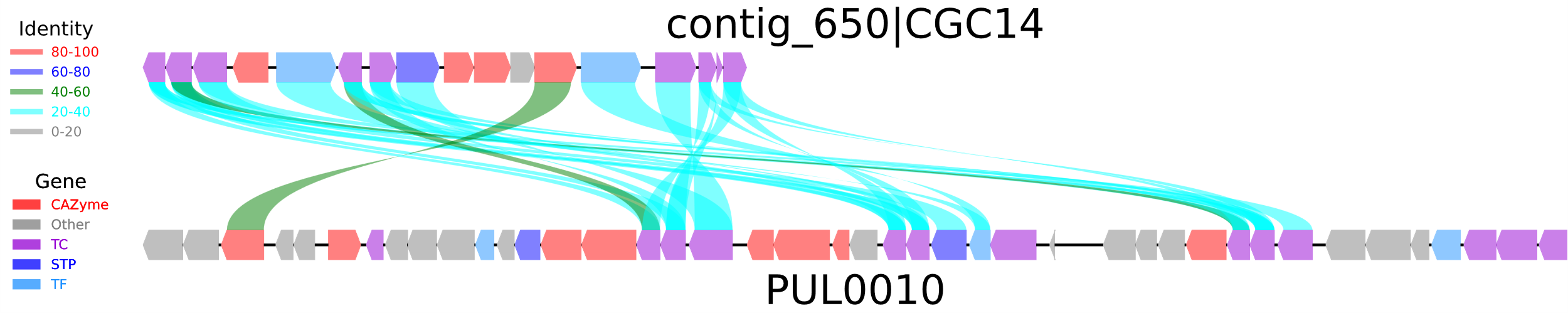  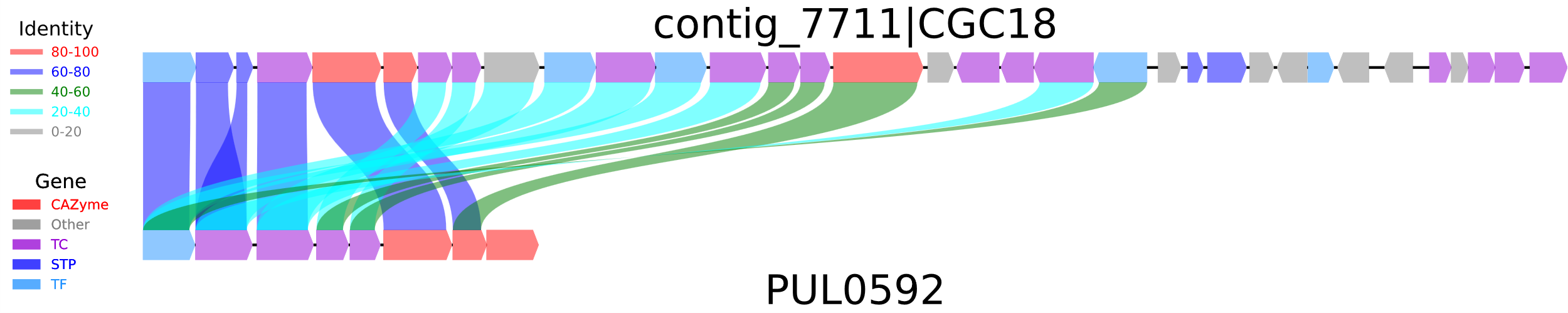  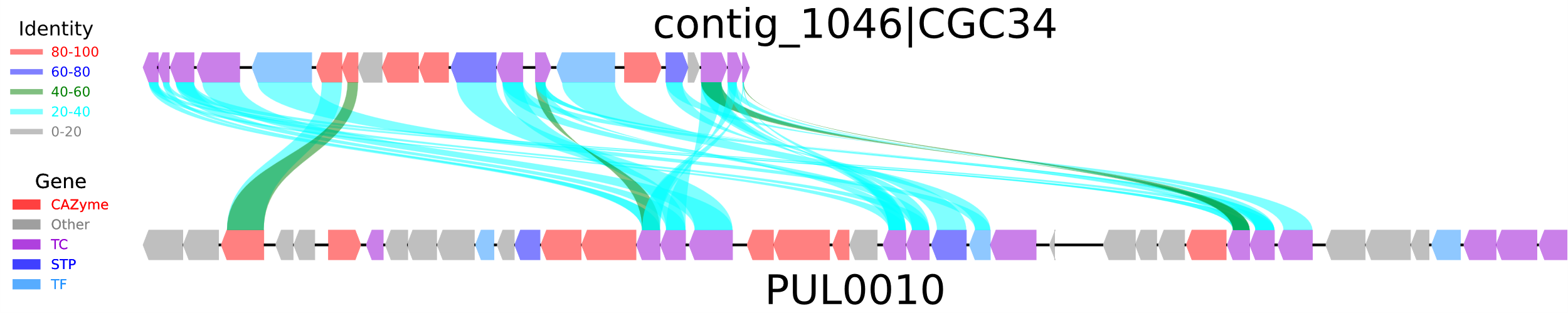 |
|  | Glucosaminoglican  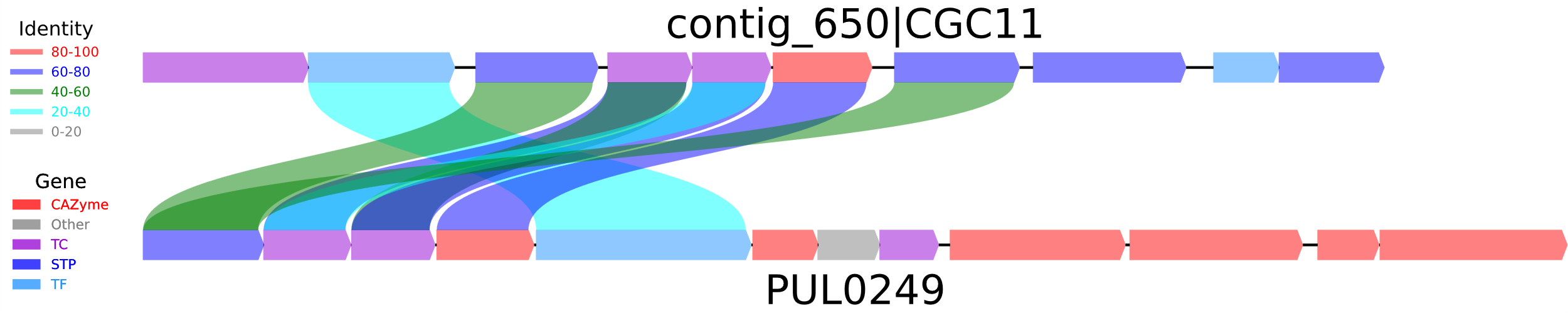 |

Figure S7. Examples of PULs structure in MAGs belonging to different genera. At the top of the alignment is the part of a contig from MAG, at the top – reference sequence from PUL database.
